# Using the Size Structure of Populations to Infer Range Dynamics and the Frequency of Recruitment

**DOI:** 10.1002/ece3.71603

**Published:** 2025-06-18

**Authors:** Jenny Ann Sweatman, J. David Aguirre, Adam N. H. Smith, Libby Liggins

**Affiliations:** ^1^ School of Natural Sciences Massey University Auckland New Zealand; ^2^ School of Biological Sciences University of Auckland Auckland New Zealand; ^3^ Sea Through Science Limited Auckland New Zealand

**Keywords:** *Centrostephanus rodgersii*, population dynamics, range shifts, recruitment, sea urchin, size structure

## Abstract

Climate change is causing shifts in the distributional ranges of species. Often, it is hard to evidence that a range shift has happened; however, because we lack time‐series data to track the distributional change of species. In Australia, *Centrostephanus rodgersii* (the Long‐spined urchin) underwent a well‐studied range extension southward to Tasmania, where it caused an ecosystem phase shift by overgrazing kelp. *Centrostephanus rodgersii* is also found in north‐eastern New Zealand (NENZ), where it could have similar impacts. Unfortunately, there are no time‐series data from which to infer population or recruitment dynamics of *C. rodgersii* in NENZ. To address this knowledge gap, we analysed the size structure (the mean and standard deviation of urchin sizes) of *C. rodgersii* populations across its geographic range using a Bayesian modelling approach. We expected that older populations with frequent recruitment would have larger mean sizes and larger standard deviations in sizes than more recently formed populations with less recruitment. We tested our model using a simulation study and population size‐structure data from across *C. rodgersii*'s extended range in Tasmania. We found a poleward decrease in mean sizes, consistent with the well‐known range extension. When applied to NENZ, we recovered a poleward decrease in mean sizes across northern populations, suggesting a recent sequential, southward colonisation in this part of the species range. Our model also showed a poleward increase in the standard deviation of sizes across NENZ, suggesting a poleward increase in the frequency of recruitment. In contrast, the southern populations of Tasmania had smaller values indicative of more limited recruitment at the leading range edge. Our study demonstrates that size‐structure data can be a valuable resource in understanding range dynamics and recruitment in the absence of time‐series data.

## Graphical abstract

Determining whether a species is undergoing a range extension is often difficult to establish without long‐term population data. Our study examined the utility of size‐structure data for studying recent range dynamics and the frequency of recruitment into populations of the Long‐spined urchin, *Centrostephanus rodgersii*. We present a conceptual model, simulation study and empirical applications of our approach.

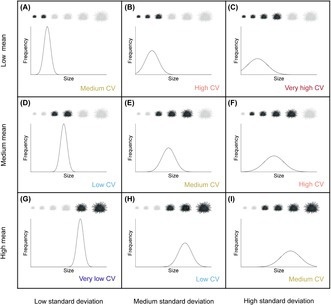

## 1 Introduction

Climate‐mediated range shifts are altering recipient ecosystems, causing widespread socio‐economic impacts (Pecl et al. 2017). Range extensions, a common type of range shift, involve a species colonising a new location, followed by population growth through increased immigration or local recruitment (Bates et al. 2014). In the marine environment, we expect to see increased immigration and colonisation of new locations as a consequence of climate‐forced changes in ocean circulation altering, and in some cases strengthening, dispersal pathways (Doney et al. 2012; Wilson et al. 2016). Furthermore, localised warming of ocean temperatures due to climate change helps species from locations with warmer ocean temperatures to survive and reproduce in new, formerly cooler locations, promoting population growth. Despite the prevalence of range extensions in the marine environment (Pinsky et al. 2020), determining whether a certain species is undergoing a range extension is often difficult to establish without long‐term population data.

Range shifts of habitat‐forming species, or species that influence the abundance and distribution of habitat‐forming species, can have dramatic impacts on the structure and function of marine communities (Doney et al. 2012; Johnson et al. 2011; Jurgens and Gaylord 2018). In many shallow subtidal marine ecosystems, kelp provides physical habitat for other species by reducing the impacts of waves and currents, preventing sediment movement and maintaining water clarity (Jones et al. 1997; Layton et al. 2019). Therefore, an increase in herbivory, either because of trophic cascades (Johnson et al. 2011; Ling et al. 2015; Shears and Babcock 2003; Tegner and Dayton 2000) or range extensions of herbivores (Johnson et al. 2011; Vergés et al. 2014; Wernberg et al. 2016), can cause the loss of the kelp forest habitat and the species the habitat supports (Johnson et al. 2011; Teagle et al. 2018). One such herbivore is the Long‐spined sea urchin (*Centrostephanus rodgersii*), which in high numbers can overgraze the kelp leaving habitats dominated by urchins and crustose algae commonly referred to as urchin barrens (Andrew 1994; Ling, Johnson, Frusher, et al. 2009; Ling, Johnson, Ridgway, et al. 2009). The impact of *C. rodgersii* has been pronounced where the species has extended its range into south‐east Australia and Tasmania. The larvae of *C. rodgersii* are capable of dispersing for 3–4 months (Huggett et al. 2005) and because of climate‐change‐driven strengthening of the East Australian Current (EAC) (Oke et al. 2019), *C. rodgersii* has been able to disperse to Tasmania (Ling, Johnson, Frusher, et al. 2009; Ling, Johnson, Ridgway, et al. 2009) on multiple occasions (Johnson et al. 2011). The EAC has also increased the sea temperature along the south‐east coast of Australia, allowing the persistence of *C. rodgersii* in Tasmania (Ling, Johnson, Frusher, et al. 2009; Ling, Johnson, Ridgway, et al. 2009). As a result of the barrens habitats created by *C. rodgersii*, Tasmanian macroalgae forests have lost at least 150 taxa (Ling 2008), and important fisheries such as blacklip abalone ( *Haliotis rubra* ) and southern rock lobster ( *Jasus edwardsii* ) have been affected (Johnson et al. 2005; Lisson 2018).

The range of *C. rodgersii* currently includes the east Australian coast, Lord Howe Island, Norfolk Island, Rangitāhua (the Kermadec archipelago) and north‐eastern New Zealand (Byrne and Andrew 2013). However, we know little about the historic range‐wide dynamics of the species. Current evidence suggests that *C. rodgersii* naturally colonised Lord Howe Island and Norfolk Island via the eastern flowing Tasman Front, an offshoot of the EAC (Oke et al. 2019), and NENZ was naturally colonised via the south‐easterly flowing East Auckland Current (EAuC) that arises from the Tasman Front (Byrne and Andrew 2020). *Centrostephanus rodgersii* was first recorded in New Zealand in 1897 (Farquhar 1897) but was subsequently removed from the faunal list on two occasions for lack of evidence. It was not until 1949, when live specimens were collected from several locations separated by more than 150 km (the Cavalli Islands, Stephen's Island, Whangaroa and Little Barrier Island), that the presence of *C. rodgersii* in New Zealand was confirmed (Fell 1949). Initial population genetic studies identified genetic similarity between *C. rodgersii* populations in New Zealand and the East Coast of Australia (Banks et al. 2007), but recent studies further suggest that New Zealand populations are a self‐sustaining meta‐population, no longer reliant on dispersal from Australia (Thomas et al. 2021). Although *C. rodgersii* is now widespread in New Zealand (spanning nearly 10° of latitude from Raoul Island, Rangitāhua in the north to Ariel Reef, Gisborne in the south) and is known to be increasing in local abundance in parts of NENZ (Balemi and Shears 2023), due to a lack of standardised survey data, we do not know the details of exactly when *C. rodgersii* extended to its current range, whether its range extension continues and which parts of the range have been most recently colonised.

*Centrostephanus rodgersii* has the potential to alter coastal processes in New Zealand's coastal ecosystem and fisheries. The dominant macroalgae in NENZ is *Ecklonia radiata*, which was the macroalgal species most impacted by *C. rodgersii* in Tasmania (Ling and Keane 2018). In New Zealand, *E. radiata* forests support high‐value fisheries of crayfish ( *J. edwardsii* , also known as southern rock lobster), pāua ( *Haliotis iris* ) and an endemic urchin, kina (*Evechinus chloroticus*). *Evechinus chloroticus* is the most abundant urchin on NENZ reefs and is generally found shallower than *C. rodgersii* (Balemi and Shears 2023); however, there is the potential these two urchin species will compete. Furthermore, *C. rodgersii* has been shown to consume sessile, habitat‐forming invertebrates (Balemi et al. 2025), and therefore can impact sensitive invertebrate‐dominated communities in New Zealand as well. For these reasons, a thorough understanding of the historical and likely future range dynamics of the species in New Zealand is a priority.

**FIGURE 1:**
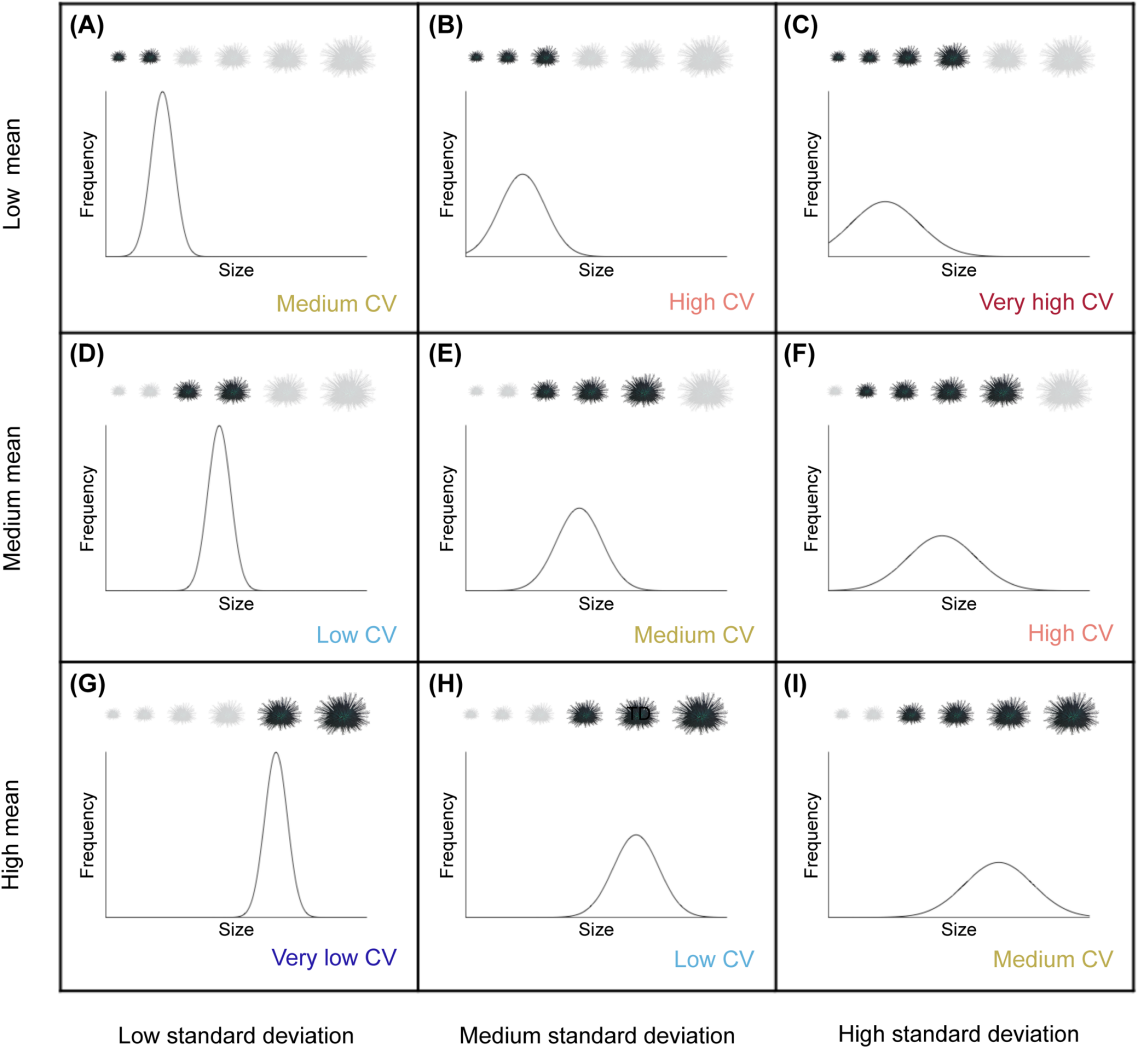
How the coefficient of variation, standard deviation and mean sizes of individuals in a population can reflect range dynamics and the frequency of recruitment across a species range. The sizes of urchins present in a population are depicted by the black urchin icons, and the sizes that are absent are in grey. The plots represent the frequency of urchin sizes in a population used to calculate the mean sizes and standard deviation of sizes in a population. Based on these measures, the coefficient of variation (CV) varies from very low (dark blue) to very high (red). In the context of a range extension, for a leading edge population, we would expect a scenario with a low mean (A), (B) and (C) because there are predominantly new, and therefore small, individuals. We would expect to see: (A) if there has only been one recent recruitment event because individuals would be predominantly within the same age cohort and therefore there would be little variation in sizes so a small standard deviation; (B) if there has been some recent recruitment because individuals would be predominantly from a few age cohorts and therefore there would be some variation in sizes so a medium standard deviation; or (C) high if the population frequently recruits because individuals would be from a range of recent age cohorts and therefore there would be a lot of variation in sizes so a large standard deviation. For a long established and regularly recruiting population at the stable centre of a species range, we would expect a scenario with a medium mean such as (F). If an established population does not have regular recruitment, or is the result of an isolated recruitment event, we would expect a scenario such as (E) and (D), respectively. For a population at the trailing edge of a species range we would expect to see a high mean (G), (H) and (I) because there are predominantly older, and therefore larger, individuals. Although the standard deviation can give us an indicator of the frequency of recruitment similar to the other scenarios, the standard deviation is potentially less informative in (G), (H) and (I) for species that reach a maximum size, because the large individuals can be from multiple age cohorts.

In the absence of time‐series survey information, the timing of colonisation and the frequency of recruitment may be reflected in the age‐class structure or size structure of a population. Size‐structure data have been used both alone and in combination with abundance and/or density to infer population dynamics for many marine invertebrates, including the red sea urchin (*Mesocentrotus franciscanus*; Tegner and Dayton 1981; Botsford et al. 1994; Morgan et al. 2001), the purple sea urchin ( *Strongylocentrotus purpuratus* ; Ebert and Russell 1988; Ebert et al. 1999; Ebert 2010), and Kellet's whelk ( *Kelletia kelletii* ; Zacherl et al. 2003) in California, USA, and the mulberry whelk (*Morula marginalba*), another marine snail (*Afrolittorina pyramidalis*), and the rose barnacle (*Tesseropora rosea*) in south‐east Australia (Hidas et al. 2010). Examining population size‐structure distributions directly provides the greatest information about the demography of the population; however, using metrics such as the mean size and standard deviation of sizes of individuals in a population allows us to statistically compare the trends across multiple populations. For instance, the mean size of individuals in a population can indicate how long a population has been established. If a population has been present for a long time, then a greater proportion of the individuals will be large adults, and the population will have a larger mean size (Figure 1G–I) relative to more recently formed populations (assuming that growth is spatially consistent). In contrast, a newly colonised population will have a greater proportion of younger, small individuals, and therefore a smaller mean size (Figure 1A–C). The standard deviation of sizes in a population can provide information about the frequency of recruitment. For instance, a low standard deviation of sizes can indicate individuals in a population are similar in size and thereby age, suggesting one recruitment event leading to one size class (Figure 1A,D,G). In contrast, a high standard deviation of sizes indicates the individuals are spread across multiple age classes, suggesting more frequent recruitment (Figure 1C,F,I).

The coefficient of variation (obtained by dividing the standard deviation by the mean) has been used to reveal how recruitment patterns vary across species ranges (Ebert and Russell 1988). A high coefficient of variation indicates the sizes of individuals are highly variable relative to the mean size and therefore suggests more frequent recruitment (Morgan et al. 2001; e.g., Figure 1B,C,F). In contrast, a small standard deviation relative to the mean, and therefore a low coefficient of variation, may indicate episodic recruitment into the population, as only a few size classes are found (e.g., Figure 1D,G,H). For example, for purple sea urchin ( *S. purpuratus* ) populations sampled from central California to central Oregon, a relationship between the coefficient of variation and the distance from a headland where upwelling occurred showed recruitment was more infrequent closer to the area of high upwelling (Ebert and Russell 1988). Black et al. (2011) also used the coefficient of variation to infer that recruitment increased with decreasing latitude for small giant clam (*Tridacna maxima*) populations within the Ningaloo Marine Park, Australia.

Although many studies have used only the coefficient of variation, consideration of the mean and standard deviation separately may yield additional insights in the context of climate‐impacted oceans. A newly established population at the leading edge of a range extension that has only young individuals from a few recruitment events would have a small standard deviation and small mean size (Figure 1A). Using the coefficient of variation alone, the dynamics of this newly established population would be indistinguishable from a long‐established population with frequent recruitment, which would have a large mean and large standard deviation (Figure 1I). Likewise, a stable population that has had multiple recruitment events over time and has a medium mean and medium standard deviation would also have a medium coefficient of variation (Figure 1E). In general terms, we may consider that the standard deviation refers to the frequency of recruitment, whereas the mean refers to the age of the population. Thus, when there are clear predictions for the expected demographic scenarios resulting from different population histories and ongoing recruitment dynamics, analysis of population size structures—including the mean, standard deviation, and coefficient of variation—can provide a more nuanced view of the population dynamics.

In Tasmania, Ling, Johnson, Frusher, et al. (2009) and Ling, Johnson, Ridgway, et al. (2009) examined the abundance and size structure of *C. rodgersii* populations distributed along the documented poleward range extension axis. The authors found an exponential decline in mean urchin size (and age inferred via growth models) with distance from the EAC and a poleward decrease in size and abundance—a pattern consistent with the documented poleward range extension. Here, we use population size‐structure data to detect similar signs of recent and/or varied colonisation events, or variable recruitment, across the range of *C. rodgersii* in NENZ. Specifically, we model trends in population size‐structure parameters (mean, standard deviation, and coefficient of variation) with latitude, where we expect smaller mean sizes in southern populations, indicative of a poleward range extension similar to what was observed in Tasmania. In addition, a high standard deviation in population sizes could indicate where populations have more frequent recruitment. To test the performance of our model, we first use simulated data to confirm if trends in both the mean and standard deviation of urchin sizes (based on measured test diameters) could be reliably recovered from the same model. Second, we modelled the size‐structure data from Tasmanian *C. rodgersii* populations to test our inferences in the case of a known range extension. Last, based on our verified approach and assumptions, we use our model to investigate the size‐structure data to infer the population history and range dynamics of *C. rodgersii* populations in New Zealand.

## 2 Materials and Methods

### 2.1 Modelling Trends in Population Size Structure

Size is determined by age and growth rate. For urchins, differences in growth rates can be due to differences in food availability or ambient temperature (Pecorino et al. 2012). In our study, we assumed that all NENZ locations have similar growth rates and used size as a surrogate for age. In support of our assumption, Ling, Johnson, Frusher, et al. (2009) and Ling, Johnson, Ridgway, et al. (2009) found that all sampled Tasmanian *C. rodgersii* populations, across a larger latitudinal gradient, had the same growth rates. Furthermore, growth models created for *C. rodgersii* at the Mokohinau Islands in NENZ were very similar to the models derived for Tasmanian populations (Pecorino et al. 2012). Additionally, the entire NENZ range of *C. rodgersii* is within one bioregion, within which algal abundance and diversity are similar (Shears et al. 2008). Nonetheless, our approach only allowed us to detect recent range dynamics, up to 10–15 years before sampling, as the ages of urchins are indistinguishable once they reach full size (~90 to 100 mm at 10–15 years; Pecorino et al. 2012).

Using a Bayesian modelling approach (using the R package ‘rethinking’ version 2.01; McElreath 2016, 2020), we created a model to detect trends in the means and standard deviations of sizes at several locations along a latitudinal gradient as shown in Equations ((1), (2), (3), (4), (5), (6), (7), (8)). Specifically, we modelled a normal distribution of individual urchin sizes ($y_{\mathrm{ij}}$) at each location *i* (Equation 1). The mean urchin size at location *i* ($\mu_{i}$) consists of the global mean urchin size ($\alpha_{\mu }$), the slope for the mean versus latitude times the latitude at location *i* ($\beta_{\mu }x_{i}$), and the error terms ($z_{\mu ,i}\tau_{\mu }$) (Equation 2). The standard deviation of urchin sizes at location *i* ($\sigma_{i}$) consists of the global location standard deviation of urchin sizes ($\alpha_{\sigma }$), the slope for standard deviation versus latitude times the latitude at location *i* ($\beta_{\sigma }x_{i}$) and the error terms ($z_{\sigma ,i}\tau_{\sigma }$) (Equation 3).

To test our modelling approach, first (see Section 2.2), we tested the model's performance using simulated size‐structure data with different trends in the mean and standard deviation of varying magnitudes. Second (see Section 2.3), we used the data from Tasmania's verified poleward range extension of *C. rodgersii* to test whether we could detect the expected poleward trend in size structure. Last (see Section 2.4), once we had verified that the model could detect the size‐structure trends we expected, we used our model to infer trends in the mean and standard deviation of size for the New Zealand populations of *C. rodgersii*.

(1) $$y_{\mathrm{ij}}∼\text{Normal}\left(\mu_{i},\sigma_{i}\right)$$

(2) $$\;\mu_{i}=\alpha_{\mu }+\beta_{\mu }x_{i}+z_{\mu ,i}\tau_{\mu }$$

(3) $$\;\sigma_{i}=\alpha_{\sigma }+\beta_{\sigma }x_{i}+z_{\sigma ,i}\tau_{\sigma }$$

(4) $$\alpha_{\mu }∼\text{Normal}\left(90,10\right)$$

(5) $$\alpha_{\sigma }∼\text{Normal}\left(15,5\right)$$

(6) $$\beta_{\mu },\beta_{\sigma }∼\text{Normal}\left(0,5\right)$$

(7) $$z_{\mu ,i},z_{\sigma ,i}∼\text{Normal}\left(0,1\right)$$

(8) $$\tau_{\mu },\tau_{\sigma }∼\text{Exponential}\left(1\right)$$

- $y_{\mathrm{ij}}$ is the size of individual j in location *i*.
- $\;\mu_{i}$ are the location means.
- $\;\sigma_{i}$ are the location standard deviations.
- $\alpha_{\mu }$ and $\alpha_{\sigma }$ are the intercepts for the mean and SD, respectively.
- $\beta_{\mu }$ and $\beta_{\sigma }$ are the slopes for the mean versus latitude and SD versus latitude, respectively.
- $x_{i}$ is latitude (centred on zero, note: higher latitudes are more negative).
- $z_{\mu ,i}$ are the standard normal deviates of the mean of location i from the regression on latitude (i.e., location‐level error in the mean).
- $z_{\sigma ,i}$ is the standard normal deviate of the standard deviation of location i from the regression on latitude (i.e., location‐level error in the standard deviation).
- $\tau_{\mu }$ is the SD of the location deviates for $\mu_{i}$.
- $\tau_{\sigma }$ is the SD of the location deviates for $\sigma_{i}$.

All our models used the same weakly informative priors (Equations (4), (5), (6), (7), (8)) leveraging values in published literature, observed data and based on knowledge of the study species as described in the Supplement. Additionally, in the Supplement, we present a sensitivity analysis of the parameters $\alpha_{\mu }$, $\alpha_{\sigma }$, $\beta_{\mu }$ and $\beta_{\sigma }$ to varying prior distributions (Table S4, Figures S3–S10).

### 2.2 Simulation Study

In the simulation study, we tested whether the model could recover changes in urchin sizes across latitudes that were of different magnitudes and directions for both the location means and location standard deviations. For each scenario, we simulated two data sets with two different sample sizes: five locations and 15 locations (where each location has a sampled population). Within each location, we simulated test diameter measurements for 30 individuals across all simulations.

**FIGURE 2:**
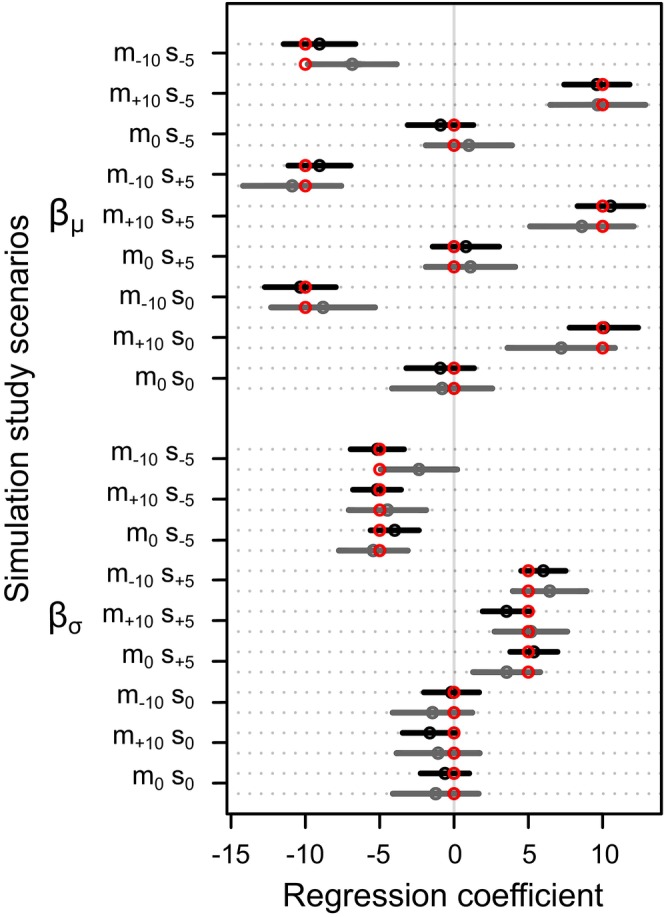
Results of the simulation study testing the Bayesian model under different scenarios. The regression coefficients $\beta_{\mu }$ (between latitude and the mean sizes of individuals at locations) and $\beta_{\sigma }$ (between latitude and the standard deviation of individual sizes at locations) were plotted for each of the nine simulated scenarios for a 15‐location dataset, shown in black, and a 5‐location dataset, shown in grey. The models are named based on the relationship between the location means and latitude (‘m_−10_’: means decreasing, $\beta_{\mu }$ = −10; ‘m_+10_’: means increasing, $\beta_{\mu }$ = 10; ‘m_0_’: means neutral, $\beta_{\mu }$ = 0) and the relationship between location standard deviations and latitude (‘v_−10_’: standard deviation decreasing, $\beta_{\sigma }$ = −5; ‘v_+10_’: standard deviation increasing, $\beta_{\sigma }$ = 5; ‘v_0_’: standard deviation neutral, $\beta_{\sigma }$ = 0). The red circles are the true values of the parameters in each simulated scenario, and the black or grey circles are the means and 95% credible intervals as estimated from the model.

Full details of the simulation study are presented in the Supplement. Briefly, we simulated nine scenarios for a combination of neutral, positive and negative relationships between urchin sizes and latitude for both the location means and location standard deviations (Figure 2). Next, we tested the sensitivity of our model to differences in the magnitude and direction of the simulated regression parameters. We simulated six additional datasets: three scenarios with different regression parameters for location means and three scenarios with different regression parameters for location standard deviations (Figures S12 and S13). Lastly, we examined if non‐linear patterns in the location means or standard deviations across latitude influenced the performance of our models. These two simulations used convex relationships between latitude and either location means or location standard deviations and no relationship with latitude and either location means or location standard deviations, respectively (Figures S14 and S15).

### 2.3 Modelling Population Size Structure for *Centrostephanus rodgersii* in Tasmania

**FIGURE 3:**
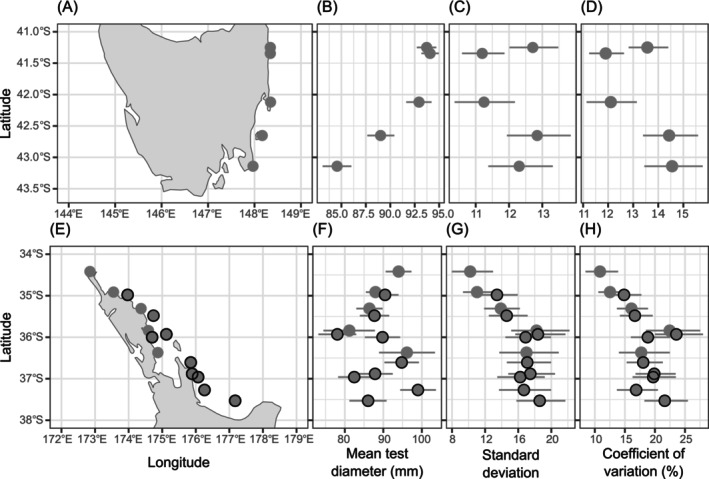
Estimated population means, standard deviations and coefficients of variation, from our model, for sampled *Centrostephanus rodgersii* populations. (A) Map of the locations sampled for *C. rodgersii* on the east coast of Tasmania; (B) Estimated mean test diameter for each location in Tasmania; (C) Estimated standard deviation of test diameters for each location in Tasmania; (D) Estimated coefficient of variation of test diameter for each location in Tasmania. (E) Map of the locations sampled for *C. rodgersii* in north‐eastern New Zealand; (F) Estimated mean test diameter for each location in north‐eastern New Zealand; (G) Estimated standard deviation of test diameters for each location in north‐eastern New Zealand; (H) Estimated coefficient of variation of test diameter for each location in north‐eastern New Zealand. Lines indicate 95% credible intervals. Locations with a black outline are offshore locations (discussed in text).

The range extension of *C. rodgersii* in Tasmania is well documented, so we used it to test the model's ability to detect a known poleward range extension using size‐structure data. Ling and Johnson collected urchins from five locations/populations in 2004 and 2005 (Figure 3D) on the east coast of Tasmania, covering the latitudinal range 43.48°S to 41.34°S (Ling and Johnson 2008, accessed from the Australian Ocean Data Network). The urchin test diameters ranged from 33 to 133 mm. Two of the locations/populations (St Helens Island and Elephant Rock) had a site for both barrens habitat and kelp habitat, which were combined here so there was only one dataset per location, each with 600 individuals sampled (details in Ling and Johnson 2009). Cape Tourville and Mistaken Cape each had 300 individuals sampled, and Fortescue had 282 individuals sampled (details in Figure S1 and Table S1).

### 2.4 Inferring Population History and Recruitment Dynamics of *Centrostephanus rodgersii* in North‐Eastern New Zealand From Models of Population Size Structure

Last, we modelled latitudinal trends in location means, location standard deviations and location coefficients of variation across the range of *C. rodgersii* in NENZ. Here, we collected maximum test diameter measurements (mm) of 647 *C. rodgersii* individuals from 14 locations (15–73 urchins per location) along the north‐eastern coast of the North Island of New Zealand (Figure 3A). We classified the locations within two nautical miles of the mainland coastline as onshore and the locations outside two nautical miles of the mainland coastline as offshore. At each location, 2–4 scuba divers descended onto rocky reef and proceeded to haphazardly collect urchins they encountered between the depths of 5–18 m. Search effort ceased once 50 urchins (or in a few cases, a greater number, depending on the conditions of collection permits) had been collected, or the dive time had reached 40 min. Urchins were brought onto the support vessel and their maximum test diameter measured using a ruler sat beneath the oral surface of the urchin. Several measurements were undertaken until at least two of the maximum measurements to the nearest millimetre taken (always by L. Liggins) were in agreement, and this measurement was recorded (usually by J. D. Aguirre). Individuals ranged from 32 to 130 mm and were collected between 2015 and 2018. The majority of locations were sampled between late 2015 and early 2016, with a few from August and September 2017 and January 2018 (details in Figure S2 and Table S2).

## 3 Results

### 3.1 Simulation Study

Overall, we found close agreement between the simulated parameters and the model estimates of these parameters across a range of realistic scenarios (Figure 2, further details in the Supplement). The models were conservative, slightly underestimating the strength of the relationship between latitude and the summary statistics when strong linear relationships were simulated (Figure S12). When a non‐linear, convex relationship between latitude and the summary statistics was simulated, and a linear model was fit to the simulated data, the relationship and the location estimates for the simulated mean urchin size were recovered, but the location estimates for the simulated standard deviations were not reliably recovered (Figure S15). Thus, the model can reliably recover a linear relationship between latitude and the location means and/or location standard deviations.

### 3.2 Modelling Population Size Structure for *Centrostephanus rodgersii* in Tasmania

Using the dataset of Tasmanian *C. rodgersii* populations, our model yielded a positive relationship between location means and standardised latitude ($\beta_{\mu }$ mean: 3.18, 95% credible interval: 1.32–4.89) meaning that high‐latitude, southern locations had smaller test diameters, consistent with a poleward range extension (Figure 3B). There was no significant trend in the location standard deviations of test diameters ($\beta_{\sigma }$ mean: −0.19, 95% credible interval: −1.29 to 0.91). Nonetheless, the two southern locations had smaller standard deviations than the two locations north of them, consistent with what would be expected at the leading edge of a range extension (Figure 3C).

### 3.3 Using Population Size Structure for *Centrostephanus rodgersii* in North‐Eastern New Zealand to Infer Population History and Recruitment Dynamics

In NENZ, the relationship between the location means and latitude was not significant ($\beta_{\mu }$ mean: 0.31, 95% credible interval: −4.27 to 3.33). However, at New Zealand's northern locations, which tended to be the onshore locations, the means decreased with latitude indicative of a poleward within‐range extension or range filling (to 36°S). At the southern latitudes, the location means were more variable and did not show any strong trend (Figure 3F). The results were clearer for the location standard deviations. We found evidence of a negative relationship between the location standard deviations and standardised latitude, ($\beta_{\sigma }$ mean: −2.54, 95% credible interval: −4.34 to −0.72), suggesting more frequent recruitment to high‐latitude, southern locations. Similarly, to the location means, the trend for location standard deviations was more apparent in the northern locations (Figure 3G). These northern locations tended to be onshore, however being above 36°S (distinguishing the northern populations from the southern populations) is still a better indicator of the trends in both location means and standard deviations than whether they were offshore or onshore.

### 3.4 Coefficients of Variation

In both New Zealand and Tasmania, the coefficient of variation followed a similar trend to the location standard deviations. The two southern locations in Tasmania had larger coefficients of variation than the northern three locations (Figure 3D). The coefficient of variation increased with latitude in the northern locations of NENZ (Figure 3H). These results suggest that there is more frequent recruitment occurring at more southern, high‐latitude locations for both Tasmania and New Zealand.

## 4 Discussion

Our study examined the utility of size‐structure data for studying recent range dynamics and the frequency of recruitment into populations when time‐series data are not available. We validated our model approach using simulated size‐structure data and verified its performance in recovering signatures of recent range extensions using size‐structure data from Tasmania, where *C. rodgersii* is documented to have undergone a poleward range extension (Johnson et al. 2005). Based on measured size‐structure data across the species' NENZ range, we found that mean sizes decreased in a poleward direction across northern locations, but not across high‐latitude, southern locations. Furthermore, our results reveal patterns of more frequent recruitment at higher latitudes in New Zealand. Below, we discuss the trends detected for *C. rodgersii* in NENZ and the likely drivers of the differences in the population histories of Tasmania and NENZ populations. We also discuss our approach with reference to other population size‐structure analyses and the limitations of such an approach.

Different trends in the mean sizes of individuals across *C. rodgersii* populations of Tasmania and New Zealand suggest different colonisation and demographic histories. In Tasmania, the mean sizes of individuals decreased in a poleward direction (Figure 3B). This pattern is in alignment with the documented population history of this species whereby there has been a poleward range extension influenced by the EAC. Ling, Johnson, Frusher, et al. (2009) and Ling, Johnson, Ridgway, et al. (2009) described an exponential decline in mean urchin age with distance from the western edge of EAC in Tasmania. The EAC travels in a poleward direction and, therefore, the range extension in Tasmania is associated with latitude. In NENZ, mean sizes appeared to decrease in a poleward direction for the northernmost locations but, in contrast to Tasmania, no such trend was apparent further south than 35.8°S. The latitudinal trend in the mean sizes of individuals across northern populations could be indicative of a within‐range extension or range filling through sequential, southward colonisation of locations within the formerly discontinuous range of *C. rodgersii* in NENZ (Figure 3B), however there were several locations around 36°S that varied in their mean sizes.

In comparison with Tasmania, where there was a consistent poleward trend of decreasing mean sizes, the presence of offshore locations and the greater number of locations and the breadth of latitudes sampled in New Zealand (4 degrees of latitude in New Zealand versus 3 degrees of latitude in Tasmania) may have influenced our ability to recover a uniform trend (Figure 3E). Accordingly, our findings likely correspond to the different physical environments, including offshore and onshore locations, found across latitudes in NENZ. Latitude itself does not drive biological processes; rather, it is the variables that are correlated with latitude in a region, such as temperature, ocean current directions, and in our case the prevalence of offshore islands, which are presumably more exposed to the EAuC (Figure 3E). Unlike the EAC in Australia, the EAuC in NENZ does not track the coastline poleward but stays offshore and more frequently meets the East Cape than more northerly locations (Stanton et al. 1997; Sutton and Bowen 2019). Therefore, a range extension influenced by ocean currents in NENZ may not be strictly linear and poleward. In fact, based on the mean sizes of urchins (Figure 3F), the offshore, higher latitude, southern locations of NENZ may have been colonised by *C. rodgersii* earlier than the lower latitude, northern locations, which were mostly onshore.

In Tasmania, we expected high‐latitude, southern locations to have smaller standard deviations of sizes due to having fewer older individuals. However, although the two southern locations had smaller standard deviations than the two locations north of them, overall, there was no significant trend (Figure 3C). In contrast, for New Zealand, there was compelling statistical evidence that the standard deviations of sizes within populations increased in a poleward direction, consistent with more frequent recruitment in higher latitude, southern populations (Figure 3G). This trend in standard deviations in New Zealand may be due to the variability of the currents affecting the recruitment of *C. rodgersii*. The strength and direction of the EAuC varies from year to year, sometimes missing lower latitude, northern locations (several of which are onshore), or only reaching them later in the summer whereas higher latitude, southern, mainly offshore locations are more consistently influenced by the EAuC (Stanton et al. 1997). The southern populations of NENZ have higher and more similar standard deviations than the northern populations (Figure 3G), potentially due to more regular recruitment within these southern, mainly offshore, populations. In comparison, given that there is limited suitable reef habitat north of Kapowairua (our most northerly location), northern NENZ populations, particularly the onshore locations, are likely limited by the frequency and/or magnitude of recruitment from the north and may also have less self‐recruitment.

The coefficient of variation for *C. rodgersii* populations in both Tasmania and NENZ was low compared to other studies (Figure 3D,H). In a study of the purple sea urchin ( *S. purpuratus* ) along the coast of California and Oregon, the coefficients of variation ranged from 18.5% to 46.7% and were considered ‘consistently poor’ and ‘consistently good’, respectively (Ebert and Russell 1988). Similarly, a study on the small giant clam ( *T. maxima* ) in Western Australia recovered coefficients of variation between 23.54% and 44.45% (Black et al. 2011). In contrast, the coefficients of variation for New Zealand populations of *C. rodgersii* ranged from 11.19% to 23.38%, and between 11.28% and 15.23% for Tasmanian populations. It may be that the low coefficients of variation we found were due to the difficulty in surveying the very small urchins in the population. Very young urchins tend to exhibit more cryptic behaviours, such as hiding in crevices (Ling and Johnson 2012; Byrne and Andrew 2020), causing them to be missed in visual surveys. Hence, our population mean sizes could be overestimated, and our population standard deviations and coefficients of variation may be underestimated. Although such a survey bias compromises our ability to compare our recovered coefficients of variation to other studies, it does not impact our study as this bias would have been consistent across all studied locations within New Zealand, and likely within Tasmania given the similar range of urchin sizes sampled.

Although we detected a poleward increase in the coefficients of variation for populations for both Tasmania and New Zealand (Figure 3D,H), this result provided little more information than the pattern in standard deviations of sizes. In the case of Tasmania, had we considered only the coefficients of variation, we may have missed important demographic insights, such as the trend in the mean size of individuals in Tasmania consistent with a poleward range extension. Furthermore, in New Zealand, the separate consideration of location mean sizes and standard deviations enabled us to infer recent population establishment at locations where the species has been known to occur for a longer period (leading to a high standard deviation). For instance, *C. rodgersii* was described as a rare occupant of the Mokohinau Islands prior to 1999 but has since increased in abundance at this location (Balemi and Shears 2023). Accordingly, our results reflect that individuals at this location have an overall low mean size, but there is a high standard deviation of sizes at this location (Figure 3), indicating that although the species has been present for many decades, there has more recently been a demographic change in the population reminiscent of a range extension (i.e., the population increase stage that follows arrival *sensu* Bates et al. 2014). Thus, separation of patterns associated with the frequency of recruitment and the duration of the population's presence is important when addressing demographic scenarios that potentially include range extensions (also suggested by Zacherl et al. 2003).

*Centrostephanus rodgersii* has been present in some locations of NENZ for at least 120 years (Farquhar 1897), much longer than the 40 years since it was recorded in Tasmania (Johnson et al. 2005). After approximately 15 years, *C. rodgersii* reaches maximum size and growth slows (estimated from Jolicoeur tag–recapture in Pecorino et al. 2012), meaning that during the period of our study, the ages of individuals recruited before approximately 2001 in New Zealand are indistinguishable by size, and so we were unable to infer range extensions and/or recruitment patterns occurring before 2001. Nonetheless, population genetic analysis suggests New Zealand populations are increasingly self‐recruiting (Thomas et al. 2021) and monitoring of a few NENZ *C. rodgersii* populations describes meaningful change in the size structure of the populations since 1999 (Balemi and Shears 2023). The initial surveys of Balemi and Shears (2023) describe populations of the Poor Knights Islands and Mokohinau Islands being dominated by a low number of large individuals, reminiscent of an old population with little recruitment. Since 2001, however, the range of urchin sizes has increased at these locations, with a higher proportion of smaller urchins indicating that the regularity of recruitment has likely been increasing, as the populations have established themselves at these locations. The study of Balemi and Shears (2023) evidences both the value of the time period over which our study was conducted across NENZ—a time of real demographic change for the species—and the value of detecting change over the last decade or so, even when a species is long‐lived and has been present in a location for a long period of time.

We have demonstrated that modelling the mean sizes and standard deviations in sizes of individuals in a population can be used to study population demography and recruitment, providing a particularly valuable tool when time‐series data are not available. In the context of the climate crisis, it is important to have a range of methods to understand the range histories of species. *Centrostephanus rodgersii* has the potential to detrimentally impact New Zealand's biodiversity and fisheries as in Tasmania (Johnson et al. 2005; Ling et al. 2008; Ling, Johnson, Frusher, et al. 2009; Ling, Johnson, Ridgway, et al. 2009; Lisson 2018; Cornwall et al. 2023). In New Zealand, *C. rodgersii* is limited to winter sea surface temperatures greater than 15°C (Pecorino, Lamare, et al. 2013), however, sea surface temperatures are predicted to increase (Law et al. 2017), which could promote a future range extension of the species (Pecorino, Barker, et al. 2013) as has already been described for other marine species in NENZ (Middleton et al. 2021, 2023). Tasmania is at a higher latitude than the New Zealand locations sampled but is warming at a much greater rate than NENZ (Shears and Bowen 2017; Sutton and Bowen 2019). Therefore, it is likely the range dynamics of *C. rodgersii* differ between Tasmania and New Zealand owing to the different periods of occupation, as well as the velocity of ocean climate change in each region. Formal time‐series surveys across *C. rodgersii*'s range are recommended to detect any future range extension, as well as increases in abundance and density beyond the approximately 15‐year limitation of our approach. However, in many cases, time and/or resources to undertake structured time‐series surveys are limited, especially as ocean climate changes are already impacting our ecosystems; in these situations, the method presented here provides a more immediate way to infer recent demographic histories, recruitment patterns, and to inform impact assessments and management decisions.

## Author Contributions

**Jenny Ann Sweatman:** conceptualization (equal), data curation (lead), formal analysis (lead), methodology (lead), visualization (lead), writing – original draft (lead). **J. David Aguirre:** conceptualization (supporting), formal analysis (equal), methodology (equal), supervision (equal), writing – original draft (supporting), writing – review and editing (supporting). **Adam N. H. Smith:** formal analysis (equal), methodology (equal), writing – review and editing (supporting). **Libby Liggins:** conceptualization (equal), data curation (supporting), formal analysis (supporting), funding acquisition (lead), methodology (supporting), project administration (lead), resources (lead), supervision (equal), writing – original draft (supporting), writing – review and editing (lead).

## Conflicts of Interest

The authors declare no conflicts of interest.

## Supporting information


Data S1.

## Data Availability

The original contributions, from New Zealand, presented in the study are publicly available in the supporting information. Publicly available datasets, from Tasmania, were analysed in this study. This data can be found here: https://metadata.imas.utas.edu.au/geonetwork/srv/eng/catalog.search#/metadata/bd5f4650‐7318‐11dd‐babd‐00188b4c0af8.

## References

[ece371603-bib-0001] Andrew, N. L. 1994. “Survival of Kelp Adjacent to Areas Grazed by Sea Urchins in New South Wales, Australia.” Australian Journal of Ecology 19: 466–472. 10.1111/j.1442-9993.1994.tb00513.x.

[ece371603-bib-0002] Balemi, C. , and N. Shears . 2023. “Emergence of the Subtropical Sea Urchin Centrostephanus Rodgersii as a Threat to Kelp Forest Ecosystems in Northern New Zealand.” Frontiers in Marine Science 10: 1224067. 10.3389/fmars.2023.1224067.

[ece371603-bib-0003] Balemi, C. A. , S. Pan , and N. T. Shears . 2025. “Broad Dietary Niche of Irrupting Subtropical Sea Urchin Exacerbates Threats to Multiple Temperate Reef Habitats.” Marine Ecology Progress Series 757: 131–144. 10.3354/meps14809.

[ece371603-bib-0004] Banks, S. , L. Piggott , J. Williamson , U. Bové , N. Holbrook , and L. Beheregaray . 2007. “Oceanic Variability and Coastal Topography Shape Genetic Structure in a Long‐Dispersing Sea Urchin.” Ecology 88: 3055–3064. 10.1890/07-0091.1. 18229840

[ece371603-bib-0005] Bates, A. E. , G. T. Pecl , S. Frusher , et al. 2014. “Defining and Observing Stages of Climate‐Mediated Range Shifts in Marine Systems.” Global Environmental Change 26: 27–38. 10.1016/j.gloenvcha.2014.03.009.

[ece371603-bib-0006] Black, R. , M. S. Johnson , J. Prince , A. Brearley , and T. Bond . 2011. “Evidence of Large, Local Variations in Recruitment and Mortality in the Small Giant Clam, *Tridacna maxima*, at Ningaloo Marine Park, Western Australia.” Marine and Freshwater Research 62: 1318–1326. 10.1071/mf11093.

[ece371603-bib-0007] Botsford, L. W. , B. D. Smith , and J. F. Quinn . 1994. “Bimodality in Size Distributions: The Red Sea Urchin *Strongylocentrotus franciscanus* as an Example.” Ecological Applications 4: 42–50. 10.2307/1942113.

[ece371603-bib-0008] Byrne, M. , and N. Andrew . 2013. “ *Centrostephanus rodgersii* .” In Sea Urchins: Biology and Ecology, edited by J. M. Lawrence , 243–256. Elsevier. 10.1016/b978-0-12-396491-5.00017-4.

[ece371603-bib-0009] Byrne, M. , and N. L. Andrew . 2020. “ *Centrostephanus rodgersii* and *Centrostephanus tenuispinus* .” In Sea Urchins: Biology and Ecology, edited by J. M. Lawrence , 379–396. Elsevier. 10.1016/b978-0-12-819570-3.00022-6.

[ece371603-bib-0060] Cornwall, C. E. , W. A. Nelson , J. D. Aguirre , et al. 2023. “Predicting the impacts of climate change on New Zealand’s seaweed‐based ecosystems.” New Zealand Journal of Botany 63, no. 1: 1–27. 10.1080/0028825x.2023.2245786.

[ece371603-bib-0010] Doney, S. C. , M. Ruckelshaus , J. E. Duffy , et al. 2012. “Climate Change Impacts on Marine Ecosystems.” Annual Review of Marine Science 4: 11–37. 10.1146/annurev-marine-041911-111611. 22457967

[ece371603-bib-0011] Ebert, T. A. 2010. “Demographic Patterns of the Purple Sea Urchin *Strongylocentrotus purpuratus* Along a Latitudinal Gradient, 1985–1987.” Marine Ecology Progress Series 406: 105–120. 10.3354/meps08547.

[ece371603-bib-0012] Ebert, T. A. , J. D. Dixon , S. C. Schroeter , et al. 1999. “Growth and Mortality of Red Sea Urchins *Strongylocentrotus franciscanus* Across a Latitudinal Gradient.” Marine Ecology Progress Series 190: 189–209. 10.3354/meps190189.

[ece371603-bib-0013] Ebert, T. A. , and M. P. Russell . 1988. “Latitudinal Variation in Size Structure of the West Coast Purple Sea Urchin: A Correlation With Headlands.” Limnology and Oceanography 33, no. 2: 286–294. 10.4319/lo.1988.33.2.0286.

[ece371603-bib-0014] Farquhar, H. 1897. “A Contribution to the History of New Zealand Echinoderms.” Zoological Journal of the Linnean Society 26: 186–198. 10.1111/j.1096-3642.1897.tb00402.x.

[ece371603-bib-0015] Fell, H. B. 1949. “The Occurrence of Australian Echinoids in New Zealand Waters.” Records of the Auckland Institute and Museum 3: 343–346. 10.1080/03036758.1975.10419371.

[ece371603-bib-0016] Hidas, E. Z. , D. J. Ayre , and T. E. Minchinton . 2010. “Patterns of Demography for Rocky‐Shore, Intertidal Invertebrates Approaching Their Geographical Range Limits: Tests of the Abundant‐Centre Hypothesis in South‐Eastern Australia.” Marine and Freshwater Research 61: 1243–1251. 10.1071/MF09317.

[ece371603-bib-0017] Huggett, M. J. , C. K. King , J. E. Williamson , and P. D. Steinberg . 2005. “Larval Development and Metamorphosis of the Australian Diadematid Sea Urchin *Centrostephanus rodgersii* .” Invertebrate Reproduction & Development 47: 197–204. 10.1080/07924259.2005.9652160.

[ece371603-bib-0018] Johnson, C. R. , S. C. Banks , N. S. Barrett , et al. 2011. “Climate Change Cascades: Shifts in Oceanography, Species' Ranges and Subtidal Marine Community Dynamics in Eastern Tasmania.” Journal of Experimental Marine Biology and Ecology 400: 17–32. 10.1016/j.jembe.2011.02.032.

[ece371603-bib-0019] Johnson, C. R. , S. Ling , J. Ross , S. Shepherd , and K. Miller . 2005. “Establishment of the Long‐Spined Sea Urchin (*Centrostephanus rodgersii*) in Tasmania: First Assessment of Potential Threats to Fisheries.” School of Zoology and Tasmanian Aquaculture and Fisheries Institute, University of Tasmania, Tasmania.

[ece371603-bib-0020] Jones, C. G. , J. H. Lawton , and M. Shachak . 1997. “Postive and Negaitve Effects of Organisms as Physical Ecosystem Engineers.” Ecology 78: 1946–1957. 10.1890/0012-9658(1997)078[1946:PANEOO]2.0.CO;2.

[ece371603-bib-0021] Jurgens, L. J. , and B. Gaylord . 2018. “Physical Effects of Habitat‐Forming Species Override Latitudinal Trends in Temperature.” Ecology Letters 21: 190–196. 10.1111/ele.12881. 29164789

[ece371603-bib-0022] Law, C. S. , G. J. Rickard , S. E. Mikaloff‐Fletcher , et al. 2017. “Climate Change Projections for the Surface Ocean Around New Zealand.” New Zealand Journal of Marine and Freshwater Research 52: 309–335. 10.1080/00288330.2017.1390772.

[ece371603-bib-0023] Layton, C. , V. Shelamoff , M. J. Cameron , M. Tatsumi , J. T. Wright , and C. R. Johnson . 2019. “Resilience and Stability of Kelp Forests: The Importance of Patch Dynamics and Environment‐Engineer Feedbacks.” PLoS One 14, no. 1: e0210220. 10.1371/journal.pone.0210220. 30682047 PMC6347235

[ece371603-bib-0024] Ling, S. D. 2008. “Range Expansion of a Habitat‐Modifying Species Leads to Loss of Taxonomic Diversity: A New and Impoverished Reef State.” Oecologia 156: 883–894. 10.1007/s00442-008-1043-9. 18481099

[ece371603-bib-0025] Ling, S. D. , and C. R. Johnson . 2008. “Data From: Growth and Age Across Range Extension Region of *Centrostephanus rodgersii* in Eastern Tasmania and Morphometric Comparison of Urchins Inhabiting Kelp Versus Barrens Habitats: Dataset 3—Allometery for Conversion Between Jaw Length and Test Diameter. IMAS Metadata Catalogue.” https://metadata.imas.utas.edu.au/geonetwork/srv/eng/catalog.search#/metadata/bd5f4650‐7318‐11dd‐babd‐00188b4c0af8.

[ece371603-bib-0026] Ling, S. D. , and C. R. Johnson . 2009. “Population Dynamics of an Ecologically Important Range‐Extender: Kelp Beds Versus Sea Urchin Barrens.” Marine Ecology Progress Series 374: 113–125. 10.3354/meps07729.

[ece371603-bib-0027] Ling, S. D. , and C. R. Johnson . 2012. “Marine Reserves Reduce Risk of Climate‐Driven Phase Shift by Reinstating Size‐ and Habitat‐Specific Trophic Interactions.” Ecological Applications 22: 1232–1245. 10.1890/11-1587.1. 22827131

[ece371603-bib-0028] Ling, S. D. , C. R. Johnson , S. Frusher , and C. K. King . 2008. “Reproductive Potential of a Marine Ecosystem Engineer at the Edge of a Newly Expanded Range.” Global Change Biology 14: 907–915. 10.1111/j.1365-2486.2008.01543.x.

[ece371603-bib-0029] Ling, S. D. , C. R. Johnson , S. D. Frusher , and K. R. Ridgway . 2009. “Overfishing Reduces Resilience of Kelp Beds to Climate‐Driven Catastrophic Phase Shift.” Proceedings of the National Academy of Sciences of the United States of America 106: 22341–22345. 10.1073/pnas.0907529106. 20018706 PMC2793314

[ece371603-bib-0030] Ling, S. D. , C. R. Johnson , K. Ridgway , A. J. Hobday , and M. Haddon . 2009. “Climate‐Driven Range Extension of a Sea Urchin: Inferring Future Trends by Analysis of Recent Population Dynamics.” Global Change Biology 15: 719–731. 10.1111/j.1365-2486.2008.01734.x.

[ece371603-bib-0031] Ling, S. D. , and J. P. Keane . 2018. Resurvey of the Long‐Spined Sea Urchin (Centrostephanus rodgersii) and Associated Barren Reef in Tasmania. Institute for Marine and Antarctic Studies, University of Tasmania, Tasmania.

[ece371603-bib-0032] Ling, S. D. , R. E. Scheibling , A. Rassweiler , et al. 2015. “Global Regime Shift Dynamics of Catastrophic Sea Urchin Overgrazing.” Philosophical Transactions of the Royal Society of London. Series B, Biological Sciences 370: 20130269. 10.1098/rstb.2013.0269.

[ece371603-bib-0033] Lisson, D. 2018. Maintaining Healthy Abalone Reef Systems on Tasmania's East Coast. Tasmanian Abalone Council Ltd.

[ece371603-bib-0034] McElreath, R. 2016. Statistical Rethinking: A Bayesian Course With Examples in R and Stan. CRC Press.

[ece371603-bib-0035] McElreath, R. 2020. “Rethinking: Statistical Rethinking Book Package. 2.01.”

[ece371603-bib-0036] Middleton, I. , J. D. Aguirre , T. Trnski , M. Francis , C. Duffy , and L. Liggins . 2021. “Introduced Alien, Range Extension or Just Visiting? Combining Citizen Science Observations and Expert Knowledge to Classify Range Dynamics of Marine Fishes.” Diversity and Distributions 27: 1278–1293. 10.1111/ddi.13273.

[ece371603-bib-0037] Middleton, I. , M. Francis , J. D. Aguirre , et al. 2023. “Occurrences of Tropical, Subtropical and Rare Marine Fishes in Aotearoa New Zealand Indicate Biodiversity Change.” Journal of Biogeography 00: 1. 10.1111/jbi.14677.

[ece371603-bib-0038] Morgan, L. E. , S. R. Wing , L. W. Botsford , C. J. Lundquist , and J. M. Diehl . 2001. “Spatial Variability in Red Sea Urchin (*Strongylocentrotus franciscanus*) Recruitment in Northern California.” Fisheries Oceanography 9: 83–98. 10.1046/j.1365-2419.2000.00124.x.

[ece371603-bib-0039] Oke, P. R. , M. Roughan , P. Cetina‐Heredia , et al. 2019. “Revisiting the Circulation of the East Australian Current: Its Path, Separation, and Eddy Field.” Progress in Oceanography 176: 102139. 10.1016/j.pocean.2019.102139.

[ece371603-bib-0040] Pecl, G. T. , M. B. Araújo , J. D. Bell , J. Blanchard , T. C. Bonebrake , and I.‐C. Chen . 2017. “Biodiversity Redistribution Under Climate Change: Impacts on Ecosystems and Human Well‐Being.” Science 355: eaai9214. 10.1126/science.aai9214. 28360268

[ece371603-bib-0041] Pecorino, D. , M. F. Barker , S. A. Dworjanyn , M. Byrne , and M. D. Lamare . 2013. “Impacts of Near Future Sea Surface pH and Temperature Conditions on Fertilisation and Embryonic Development in *Centrostephanus rodgersii* From Northern New Zealand and Northern New South Wales, Australia.” Marine Biology 161: 101–110. 10.1007/s00227-013-2318-1.

[ece371603-bib-0042] Pecorino, D. , M. D. Lamare , and M. F. Barker . 2012. “Growth, Morphometrics and Size Structure of the Diadematidae Sea Urchin *Centrostephanus rodgersii* in Northern New Zealand.” Marine and Freshwater Research 63: 624–634. 10.1071/mf12040.

[ece371603-bib-0044] Pecorino, D. , M. D. Lamare , M. F. Barker , and M. Byrne . 2013. “How Does Embryonic and Larval Thermal Tolerance Contribute to the Distribution of the Sea Urchin *Centrostephanus rodgersii* (Diadematidae) in New Zealand?” Journal of Experimental Marine Biology and Ecology 445: 120–128. 10.1016/j.jembe.2013.04.013.

[ece371603-bib-0045] Pinsky, M. L. , R. L. Selden , and Z. J. Kitchel . 2020. “Climate‐Driven Shifts in Marine Species Ranges: Scaling From Organisms to Communities.” Annual Review of Marine Science 12: 153–179. 10.1146/annurev-marine-010419-010916. 31505130

[ece371603-bib-0046] Shears, N. T. , and R. C. Babcock . 2003. “Continuing Trophic Cascade Effects After 25 Years of No‐Take Marine Reserve Protection.” Marine Ecology Progress Series 246: 1–16. 10.3354/meps246001.

[ece371603-bib-0047] Shears, N. T. , and M. M. Bowen . 2017. “Half a Century of Coastal Temperature Records Reveal Complex Warming Trends in Western Boundary Currents.” Scientific Reports 7: 14527. 10.1038/s41598-017-14944-2. 29109445 PMC5674067

[ece371603-bib-0048] Shears, N. T. , F. Smith , R. C. Babcock , C. A. Duffy , and E. Villouta . 2008. “Evaluation of Biogeographic Classification Schemes for Conservation Planning: Application to New Zealand's Coastal Marine Environment.” Conservation Biology 22: 467–481. 10.1111/j.1523-1739.2008.00882.x. 18294299

[ece371603-bib-0049] Stanton, B. R. , P. J. H. Sutton , and S. M. Chiswell . 1997. “The East Auckland Current, 1994–95.” New Zealand Journal of Marine and Freshwater Research 31: 537–549. 10.1080/00288330.1997.9516787.

[ece371603-bib-0050] Sutton, P. J. H. , and M. Bowen . 2019. “Ocean Temperature Change Around New Zealand Over the Last 36 Years.” New Zealand Journal of Marine and Freshwater Research 53: 305–326. 10.1080/00288330.2018.1562945.

[ece371603-bib-0052] Teagle, H. , D. A. Smale , and D. Schoeman . 2018. “Climate‐Driven Substitution of Habitat‐Forming Species Leads to Reduced Biodiversity Within a Temperate Marine Community.” Diversity and Distributions 24: 1367–1380. 10.1111/ddi.12775.

[ece371603-bib-0053] Tegner, M. J. , and P. K. Dayton . 1981. “Population Structure, Recruitment and Mortality of Two Sea Urchins (*Strongylocentrotus franciscanus* and *S. purpuratus* ) in a Kelp Forest.” Marine Ecology Progress Series 5: 255–268. 10.3354/meps005255.

[ece371603-bib-0054] Tegner, M. J. , and P. K. Dayton . 2000. “Ecosystem Effects of Fishing in Kelp Forest Communities.” ICES Journal of Marine Science 57: 579–589. 10.1006/jmsc.2000.0715.

[ece371603-bib-0055] Thomas, L. J. , L. Liggins , S. C. Banks , et al. 2021. “The Population Genetic Structure of the Urchin *Centrostephanus rodgersii* in New Zealand With Links to Australia.” Marine Biology 168: 1–11. 10.1007/s00227-021-03946-4.

[ece371603-bib-0056] Vergés, A. , P. D. Steinberg , M. E. Hay , et al. 2014. “The Tropicalization of Temperate Marine Ecosystems: Climate‐Mediated Changes in Herbivory and Community Phase Shifts.” Proceedings of the Royal Society B: Biological Sciences 281: 20140846. 10.1098/rspb.2014.0846. PMC4100510 25009065

[ece371603-bib-0057] Wernberg, T. , S. Bennett , R. C. Babcock , et al. 2016. “Climate‐Driven Regime Shift of a Temperate Marine Ecosystem.” Science 353: 169–172. 10.1126/science.aad8745. 27387951

[ece371603-bib-0058] Wilson, L. J. , C. J. Fulton , A. M. Hogg , K. E. Joyce , B. T. M. Radford , and C. I. Fraser . 2016. “Climate‐Driven Changes to Ocean Circulation and Their Inferred Impacts on Marine Dispersal Patterns.” Global Ecology and Biogeography 25: 923–939. 10.1111/geb.12456.

[ece371603-bib-0059] Zacherl, D. , S. D. Gaines , and S. I. Lonhart . 2003. “The Limits to Biogeographical Distributions: Insights From the Northward Range Extension of the Marine Snail, *Kelletia kelletii* (Forbes, 1852).” Journal of Biogeography 30: 913–924. 10.1046/j.1365-2699.2003.00899.x.

