# Supplementary material for: Using the Size Structure of Populations to Infer Range Dynamics and the Frequency of Recruitment
Source: Ecol Evol. 2025 Jun 18;15(6):e71603. doi: 10.1002/ece3.71603 (PMC12176499; doi:10.1002/ece3.71603)
Supplement: Supplementary file 1 — Data S1. [file ECE3-15-e71603-s001.pdf]

Supporting information

Using the size structure of populations to infer range dynamics and the frequency of recruitment

Jenny Ann Sweatman, J. David Aguirre, Adam N. H. Smith, Libby Liggins

Contents:

1. Supplementary data

1.1 Raw data for Tasmania
  1.1.1 Histograms of size structure
  1.1.2 Summary statistics
  1.1.3 Raw data
1.2 Raw data from north-eastern New Zealand
  1.2.1 Histograms of size structure
  1.2.2 Summary statistics
  1.2.3 Raw data

2. Description of the Bayesian model and sensitivity analysis of prior distributions

2.1 Model summary
2.2 Summary of priors choice
  2.2.1 Overall intercept for the mean test diameter α_μ
  2.2.2 Overall intercept for the standard deviation of the test diameter α_σ
  2.2.3 Slope of the relationship between the mean size and latitude β_μ
  2.2.4 Slope of the relationship between the standard deviation and latitude β_σ
2.3 Tables and Figures

3. Simulation study examining relationships between latitude and location means and standard deviations of urchin test diameters

3.1 Background for simulation study
3.2 Summary of results
3.3 Recovering linear relationships
3.4 Testing different magnitudes of the regression parameters
3.5 Recovering location means and location standard deviations
3.6 Testing the model with non-linear relationships between latitude and the location summary statistics

3.7 Supplementary tables: Outputs from models

4. Literature cited

1. Supplementary data

1.1 Raw data for Tasmania

1.1.1 Histograms for Tasmania

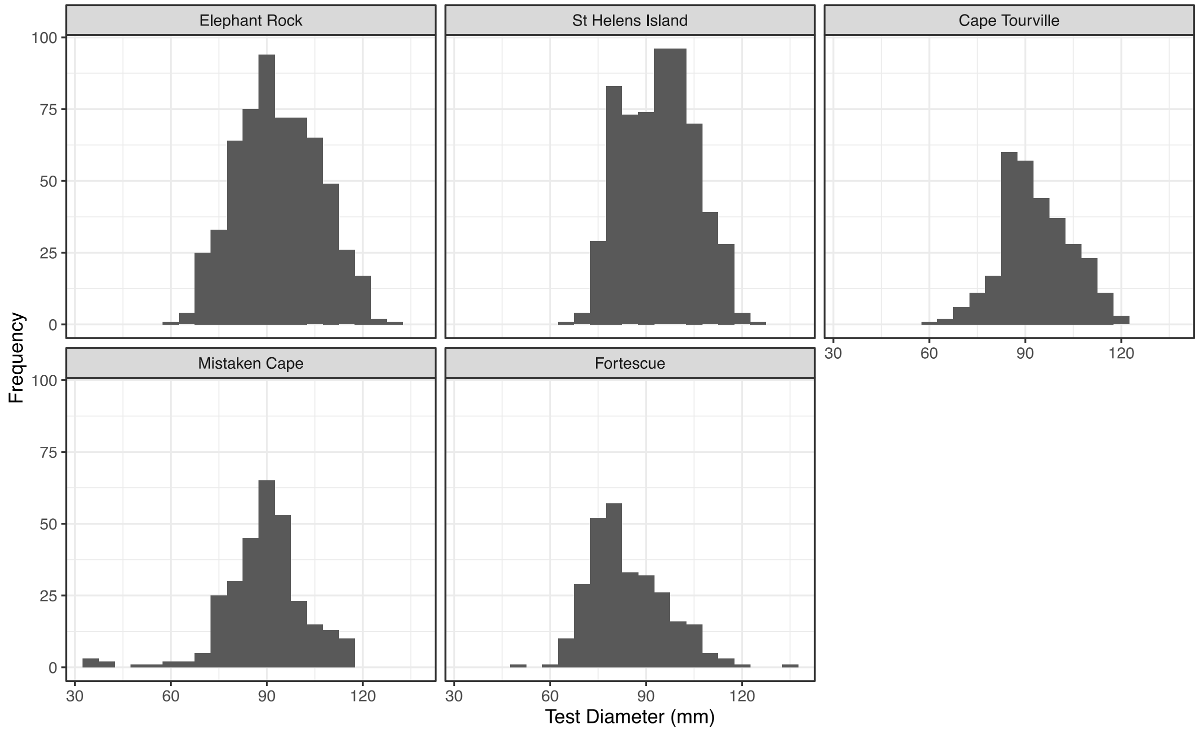

Figure S1: Histograms of the test diameter sizes, grouping into 5mm for each Tasmanian location (ordered from North to South from left to right).

1.1.2 Summary statistics for Tasmania

Population	Mean	Standard deviation	Coefficient of variation	Latitude	Longitude	Number of individuals
Elephant Rock	93.60	12.84	0.14	-41.25	148.34	600
St Helens Island	94.05	11.08	0.12	-41.34	148.34	598
Cape Tourville	93.31	10.98	0.12	-42.12	148.35	300
Mistaken Cape	89.17	13.01	0.15	-42.65	148.17	295
Fortescue	84.21	12.22	0.15	-43.14	147.97	282

Table S1: Summary statistics for Tasmania. The mean is the mean test diameter for each location, the standard deviation is the standard deviation of test diameters for each location, and the coefficient of variation is the standard deviation divided by the mean for each location. The latitude and longitude are given in decimal degrees.

1.1.3 Raw data

Publicly available datasets, from Tasmania, were used in this study. This data can be found here: https://metadata.imas.utas.edu.au/geonetwork/srv/eng/catalog.search#/metadata/bd5f4650-7318-11dd-babd-00188b4c0af8

1.2 Raw data for north-eastern New Zealand

1.2.1 Histograms for north-eastern New Zealand

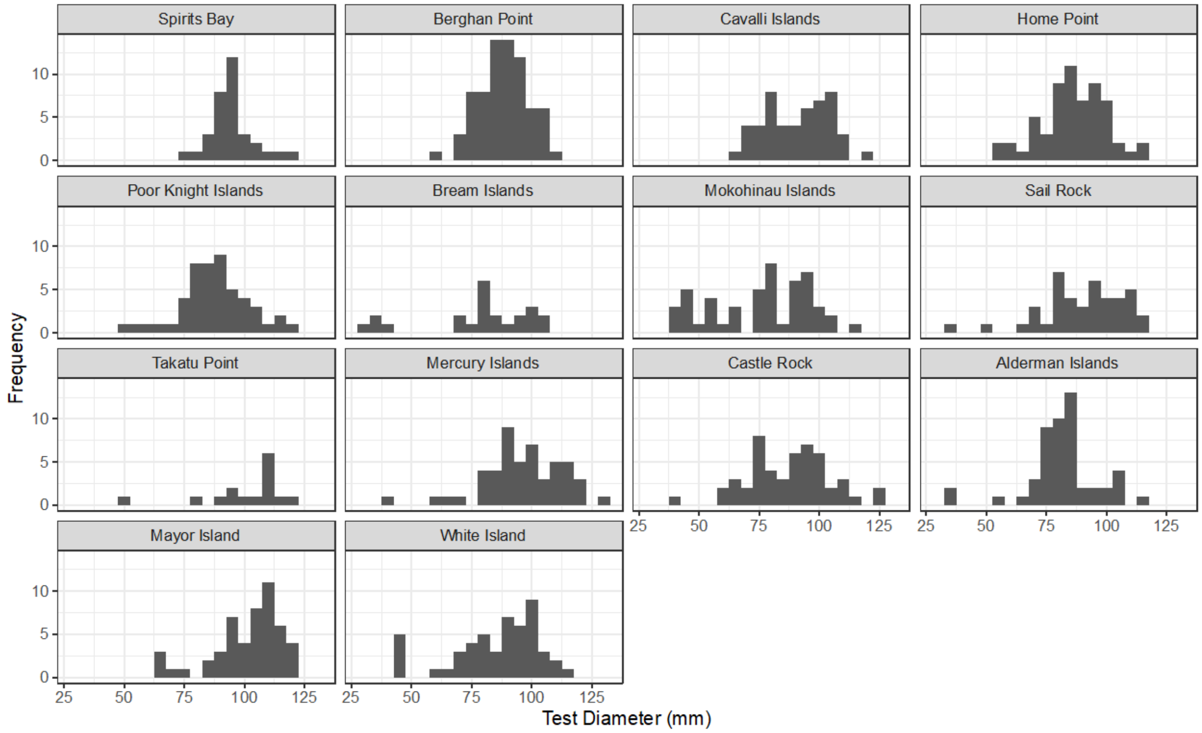

Figure S2: Histograms of the test diameter sizes, grouping into 5mm for each north-eastern New Zealand location (ordered from North to South from left to right).

1.2.2 Summary statistics for north-eastern New Zealand

Population	Mean	Standard deviation	Coefficient of variation	Latitude	Longitude	Number of individuals	Collection dates	Onshore or offshore
Spirits Bay	94.58	9.02	0.10	-34.42	172.85	33	25/03/18	Onshore
Berghan Point	87.95	10.28	0.12	-34.91	173.55	73	12/01/16, 13/01/16, 13/01/15	Onshore
Cavalli Islands	90.68	13.29	0.15	-34.98	173.97	50	13/01/16	Offshore

Home Point	86.13	13.59	0.16	-35.31	174.37	61	11/01/16, 12/01/16, 12/01/15	Onshore
Poor Knight Islands	87.56	14.48	0.17	-35.48	174.74	50	31/03/17	Offshore
Bream Islands	77.70	22.43	0.29	-35.84	174.59	23	14/01/16, 12/02/16	Onshore
Mokohinau Islands	75.68	20.52	0.27	-35.93	175.12	50	26/03/16	Offshore
Sail Rock	90.00	17.77	0.20	-36.00	174.70	42	10/01/16	Offshore
Takatu Point	100.73	17.31	0.17	-36.37	174.87	15	28/04/16	Onshore
Mercury Islands	95.92	16.93	0.18	-36.61	175.85	50	05/05/16	Offshore
Castle Rock	87.60	17.12	0.20	-36.88	175.89	50	02/02/16	Offshore
Alderman Islands	81.28	14.82	0.18	-36.96	176.07	50	03/02/16	Offshore
Mayor Island	100.92	14.84	0.15	-37.27	176.26	50	28/03/16	Offshore
White Island	85.36	17.91	0.21	-37.53	177.17	50	27/03/16	Offshore

Table S2: Summary statistics for north-eastern New Zealand. The mean is the mean test diameter for each location, the standard deviation is the standard deviation of test diameters for each location, and the coefficient of variation is the standard deviation divided by the mean for each location. The latitude and longitude are given in decimal degrees. The collection dates are the dates which samples from each location were collected. The onshore or offshore refers to whether a location was within two nautical miles of the mainland coastline (onshore) or over two nautical miles from the mainland coastline (offshore).

1.2.3 Raw data

Population	Test sizes
Spirits Bay	87, 95, 94, 103, 121, 97, 113, 91, 74, 78, 93, 91, 102, 103, 109, 89, 89, 95, 91, 94, 97, 87, 92, 93, 102, 92, 83, 95, 98, 93, 94, 95, 91
Berghan Point	105, 105, 89, 84, 105, 94, 111, 75, 106, 78, 96, 100, 90, 74, 89, 77, 95, 93, 94, 83, 72, 91, 89, 95, 100, 103, 85, 87, 100, 92, 84, 89, 79, 93, 77, 83, 76, 78, 80, 74, 84, 90, 84, 74, 80, 85, 69, 69, 73, 85, 60, 90, 95, 90, 85, 90, 96, 87, 87, 95, 87, 78, 79, 94, 100, 88, 90, 98, 78, 105, 100, 95, 90
Cavalli Islands	80, 102, 78, 80, 110, 98, 104, 111, 105, 86, 106, 88, 84, 74, 88, 80, 83, 82, 82, 107, 79, 100, 72, 97, 108, 70, 77, 75, 75, 106, 96, 89, 119, 70, 101, 98, 104, 103, 94, 80, 94, 93, 98, 100, 105, 88, 84, 97, 63, 71
Home Point	80, 80, 80, 88, 60, 69, 55, 58, 81, 78, 70, 102, 81, 90, 83, 100, 78, 95, 70, 64, 85, 106, 84, 116, 96, 94, 97, 54, 113, 87, 98, 82, 83, 100, 76, 111, 103, 92, 90, 85, 87, 84, 92, 77, 94, 70, 98, 94, 85, 74, 80, 90, 85, 100, 95, 100, 95, 95, 85, 90, 70

Poor Knight Islands	116, 115, 120, 111, 84, 86, 80, 87, 93, 91, 85, 94, 93, 105, 103, 92, 78, 79, 103, 89, 48, 81, 99, 87, 97, 74, 76, 89, 85, 60, 100, 81, 92, 86, 102, 95, 89, 100, 54, 67, 74, 88, 90, 83, 78, 80, 75, 92, 81, 71
Bream Islands	38, 82, 98, 78, 82, 105, 79, 104, 35, 102, 83, 93, 98, 70, 77, 93, 71, 84, 32, 89, 80, 34, 80
Mokohinau Islands	63, 38, 78, 104, 94, 82, 46, 76, 102, 97, 82, 44, 78, 95, 57, 84, 42, 57, 75, 88, 65, 45, 74, 93, 93, 78, 80, 98, 90, 90, 53, 93, 53, 93, 64, 91, 77, 45, 107, 39, 48, 78, 44, 77, 91, 59, 81, 113, 100, 90
Sail Rock	33, 66, 68, 102, 106, 80, 112, 107, 82, 97, 52, 84, 116, 83, 78, 78, 93, 108, 111, 83, 72, 106, 88, 96, 102, 78, 109, 92, 98, 112, 97, 105, 117, 96, 73, 79, 96, 69, 85, 89, 100, 82
Takatu Point	110, 105, 108, 97, 80, 99, 110, 115, 110, 110, 50, 90, 118, 112, 97
Mercury Islands	115, 78, 70, 110, 130, 88, 105, 90, 90, 90, 98, 118, 115, 98, 67, 98, 102, 38, 87, 94, 85, 110, 80, 90, 104, 100, 92, 90, 109, 78, 85, 90, 95, 115, 115, 95, 120, 109, 98, 58, 115, 98, 105, 82, 87, 122, 108, 95, 90, 95
Castle Rock	105, 109, 60, 113, 97, 105, 70, 77, 73, 68, 95, 95, 90, 90, 100, 100, 90, 100, 123, 110, 98, 73, 94, 81, 102, 83, 125, 79, 110, 75, 95, 67, 73, 92, 75, 92, 92, 74, 84, 40, 77, 62, 102, 82, 78, 66, 85, 97, 94, 63
Alderman Islands	77, 85, 82, 90, 73, 78, 80, 73, 83, 85, 35, 93, 78, 89, 76, 84, 87, 83, 86, 72, 85, 73, 104, 103, 78, 82, 73, 73, 66, 35, 83, 68, 103, 100, 85, 76, 83, 116, 102, 87, 94, 76, 80, 106, 80, 78, 68, 84, 78, 56
Mayor Island	115, 103, 95, 106, 93, 108, 66, 97, 93, 90, 110, 88, 74, 120, 102, 63, 112, 98, 69, 110, 111, 115, 105, 99, 116, 94, 64, 103, 114, 108, 84, 88, 120, 94, 116, 122, 107, 104, 112, 98, 105, 94, 108, 120, 109, 86, 106, 110, 114, 108
White Island	98, 100, 74, 82, 72, 72, 45, 88, 104, 113, 47, 90, 97, 72, 109, 81, 84, 93, 60, 83, 46, 47, 91, 92, 102, 100, 78, 100, 99, 94, 89, 95, 104, 73, 81, 111, 85, 76, 88, 97, 44, 78, 107, 76, 98, 92, 67, 94, 98, 102

Table S3: Raw test diameters for north-eastern New Zealand. Each test diameter measurement, in mm, used in this study is listed by the population it was collected from.

2. Description of the Bayesian model and sensitivity analysis of prior distributions

Here, we describe the Bayesian model we used to estimate the mean and standard deviation of urchin sizes (measured test diameters) at different latitudes along the coasts of north-eastern New Zealand (NENZ) and Tasmania. We give details of the prior distributions used and additional analyses assessing the sensitivity of the posterior distributions to different choices of priors. Below is a description of our 'base case' model used in the article, with prior distributions chosen based on knowledge of the study species and initial predictive checks.

Our sensitivity analyses explored the influence of: increasing or decreasing the mean of the normal prior distribution for (*2.2.1*) α_μ, the overall intercept of the mean and (*2.2.2*) α_σ, the standard deviation of test diameters; and increasing the standard deviations of the normal prior for (*2.2.3*) β_μ, the slope of the relationship between the mean test diameter and latitude and (*2.2.4*) β_σ, the slope of the relationship between the standard deviation and latitude (as shown in Table S4). We refit the model to each dataset (NENZ and Tasmania) using each prior distribution in turn. We then compared the posterior distributions of the parameters among the fits using different priors to determine whether our priors strongly affected our inferences (Fig. S3-10). We then determined sensible priors based on both what this prior would mean in the context of urchin sizes across locations (Table S4) and our sensitivity analyses.

Overall, the posterior distributions of the parameters were quite consistent between the base case and sensitivity prior distributions, indicating that our choice of priors were not having undue influence on our results. The two exceptions were the overall intercepts for the mean and standard deviation (α_μ and α_σ). The posterior distributions for these parameters did vary with different priors; however, the priors we used in the base case were realistic and those used in the sensitivity analysis were not, and, at any rate, the values of these parameters were not critical to the results in our paper.

More detailed descriptions of the model, priors, and results of the sensitivity analysis follow.

2.1 Model summary

$$y_{ij} \sim Normal(\mu_i \, , \, \sigma_i) \tag{1}$$

$$\mu_i = \alpha_\mu + \beta_\mu x_i + z_{\mu,i}\tau_\mu \tag{2}$$

$$\sigma_i = \alpha_\sigma + \beta_\sigma x_i + z_{\sigma,i}\tau_\sigma \tag{3}$$

$$\alpha_\mu \sim Normal(90,10) \tag{4}$$

$$\alpha_\sigma \sim Normal(15,5) \tag{5}$$

$$\beta_\mu \, , \, \beta_\sigma \sim Normal(0,5) \tag{6}$$

$$z_{\mu,i} \, , \, z_{\sigma,i} \sim Normal(0,1) \tag{7}$$

$$\tau_\mu \, , \, \tau_\sigma \sim Exponential(1) \tag{8}$$

- y_{ij} *is the size of individual j in location i*
- μ_i *are the location means*
- σ_i *are the location standard deviations*
- α_μ *and* α_σ *are the intercepts for the mean and SD, respectively*
- β_μ *and* β_σ *are the slopes for the mean vs latitude and SD vs latitude, respectively*
- x_i *is latitude (centred on zero, note: higher latitudes are more negative)*
- $z_{\mu,i}$ *are the standard normal deviates of the mean of location i from the regression on latitude (i.e., location-level error in the mean)*
- $z_{\sigma,i}$ *is the standard normal deviate of the standard deviation of location i from the regression on latitude (i.e., location-level error in the standard deviation)*
- τ_μ *is the SD of the location deviates for* μ_i
- τ_σ *is the SD of the location deviates for* σ_i

2.2 Summary of priors choice

2.2.1 Overall intercept for the mean test diameter α_μ

The sensitivity prior N(200, 10) resulted in a higher posterior distribution of α_μ compared to the base prior for both NENZ (Fig. S3) and Tasmania (Fig. S4). The sensitivity prior N(30, 10) had a slightly lower posterior distribution of α_μ compared to the base prior for both NENZ (Fig. S3) and Tasmania (Fig. S4). However, given the range of sizes of sampled urchins across NENZ and Tasmania was 32-133mm the posterior distribution scenarios for N(200, 10) and N(30, 10) described in Table S4 are not sensible in our context and therefore we are confident in our base prior choice.

2.2.2 Overall intercept for the standard deviation of the test diameter α_σ

The sensitivity prior N(30, 5) resulted in a slightly higher posterior distribution of α_σ compared to the base prior for NENZ (Fig. S5) and very slightly for Tasmania (Fig. S6). The sensitivity prior N(5, 5) resulted in a slightly lower posterior distribution of α_σ compared to the base prior for NENZ (Fig. S5) and very slightly for Tasmania (Fig. S6). Since there was only a small change and the base prior has the most appropriate posterior distribution scenario for our context (Table S4), we are confident in our base prior choice.

2.2.3 Slope of the relationship between the mean size and latitude β_μ

The sensitivity priors N(0, 15) did not change the posterior distribution of $\beta_\mu$compared to the base prior for both NENZ (Fig. S7) and Tasmania (Fig. S8). Therefore, our prior choice is likely not impacting the results and so we confident in our base prior choice.

2.2.4 Slope of the relationship between the standard deviation and latitude β_σ

The sensitivity priors N(0, 10) did not change the posterior distribution of β_σ compared to the base prior for both NENZ (Fig. S9) and Tasmania (Fig. S10). Therefore, our prior choice is likely not impacting the results and so we confident in our base prior choice.

2.3 Tables and Figures

Parameter	Prior distribution	Posterior distribution scenario
α_μ	N(90,10)<br><br>(base)	Mean size at a location at latitude zero between 70mm and 110mm.
	N(200,10)<br><br>(sensitivity)	Mean size at a location at latitude zero between 180mm and 220mm.
	N(30,10)<br><br>(sensitivity)	Mean size at a location at latitude zero between 10mm and 50mm.
α_σ	N(15,5)<br><br>(base)	The range of sizes at a location at latitude zero vary by between a difference of 20mm (80mm to 100mm) and a difference of 100mm (40mm to 140mm).
	N(30,5)<br><br>(sensitivity)	The range of sizes at a location at latitude zero vary by between a difference of 80mm (50mm to 130mm) and a difference of 160mm (10mm to 170mm).
	N(5,5)<br><br>(sensitivity)	The range of sizes at a location at latitude zero vary by between no difference in sizes (all 90mm) and a difference of 60mm (60mm to 120mm).
β_μ	N(0,5)<br><br>(base)	NENZ: The mean size at a location can vary up to from 70mm to 110mm across +/-2 degrees of latitude.<br><br>Tas: The mean size at a location can vary up to from 75mm to 105mm across +/-1.5 degrees of latitude.
	N(0,15)<br><br>(sensitivity)	NENZ: The mean size at a location can vary up to from 30mm to 150mm across +/-2 degrees of latitude.<br><br>Tas: The mean size at a location can vary up to from 45mm to 135mm at across +/-1.5 degrees of latitude.
β_σ	N(0,5)<br><br>(base)	NENZ: The range of sizes at a location can vary from no difference to a difference of 140mm across +/-2 degrees of latitude.<br><br>Tas: The range of sizes at a location can vary from no difference to a difference of 120mm across +/-1.5 degrees of latitude.
	N(0,10)<br><br>(sensitivity)	NENZ: The range of sizes at a location can vary from no difference to a difference of 220mm across +/-2 degrees of latitude.<br><br>Tas: The range of sizes at a location can vary from no difference to a range of 180mm across +/-1.5 degrees of latitude.

Table S4: Each of the base and sensitivity scenarios for the parameters: α_μ, α_σ, β_μ, and β_σ. The posterior distribution scenario explains what this prior would mean in the context of urchin sizes in locations. We used +/- two standard deviations to describe the likely ranges. NENZ is north-eastern New Zealand; Tas is Tasmania.

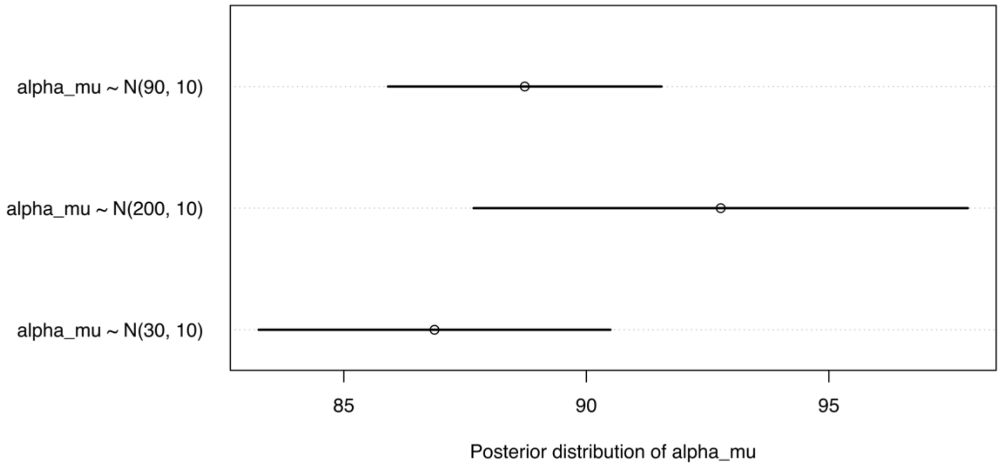

Figure S3: Testing the sensitivity of the posterior distribution to different choices of prior distribution for *alpha_mu* (α_μ) for the north-eastern New Zealand data. The points and lines show the means and 95% credible intervals of the posterior distribution of the parameter $\alpha_\mu$obtained with each of the prior distributions indicated on the y-axis. (The y-axis shows the changes that were made to the model described in Table S4).

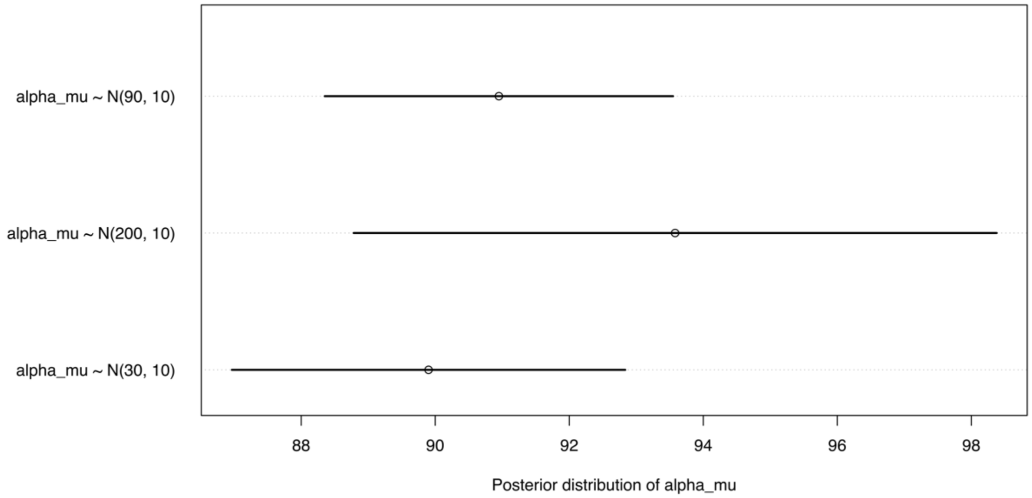

Figure S4: Testing the sensitivity of the posterior distribution to different choices of prior distribution for *alpha_mu* (α_μ) for the Tasmanian data. The points and lines show the means and 95% credible intervals of the posterior distribution of the parameter α_μ obtained with each of the prior distributions indicated on the y-axis. (The y-axis shows the changes that were made to the model described in Table S4).

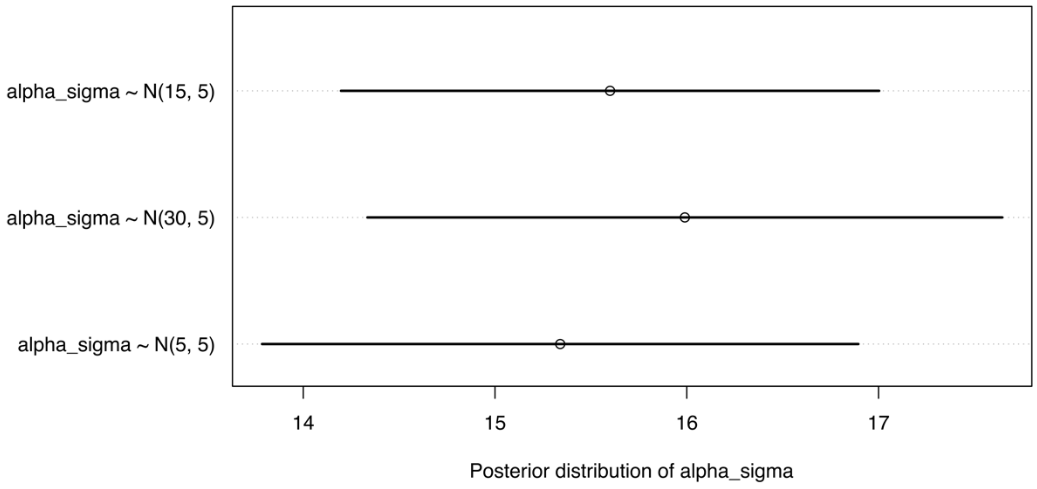

Figure S5: Testing the sensitivity of the posterior distribution to different choices of prior distribution for *alpha_sigma* (α_σ) for the north-eastern New Zealand data. The x-axis shows the 95% credible interval of the α_σ for each of the models described on the y-axis. (The y-axis shows the changes that were made to the model described in Table S4).

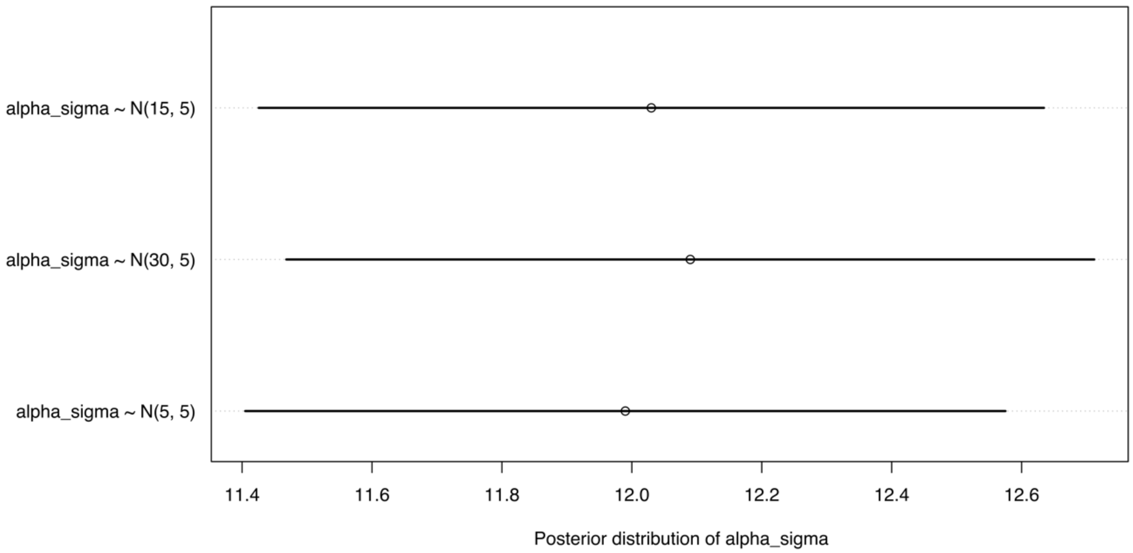

Figure S6: Testing the sensitivity of the posterior distribution to different choices of prior distribution for *alpha_sigma* (α_σ) for the Tasmanian data. The x-axis shows the 95% credible interval of the α_σ for each of the models described on the y-axis. (The y-axis shows the changes that were made to the model described in Table S4).

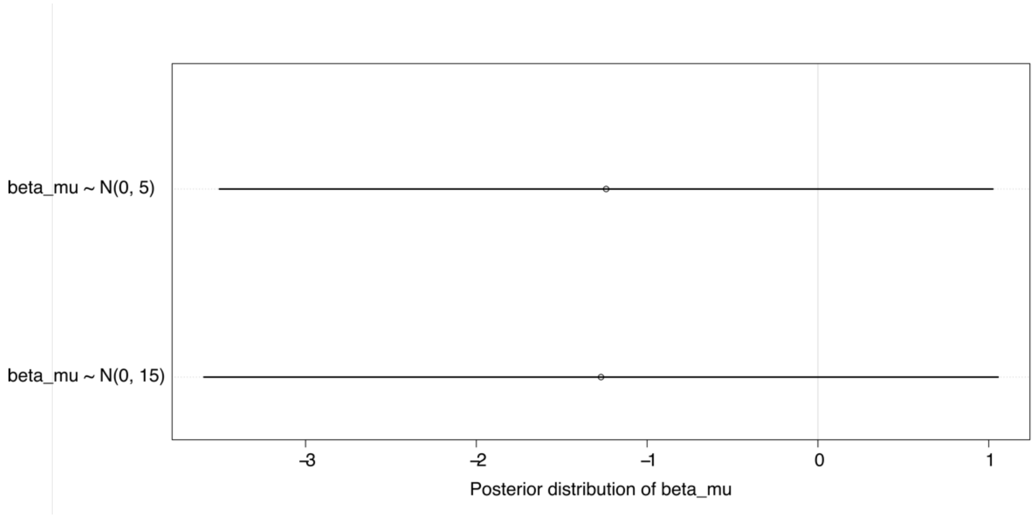

Figure S7: Testing the sensitivity of the posterior distribution to a weaker prior distribution for the parameter *beta_mu* (β_μ) for the north-eastern New Zealand data. The points and lines show the means and 95% credible intervals of the posterior distribution of the parameter β_μ obtained with each of the prior distributions indicated on the y-axis. (The y-axis shows the changes that were made to the model described in Table S4).

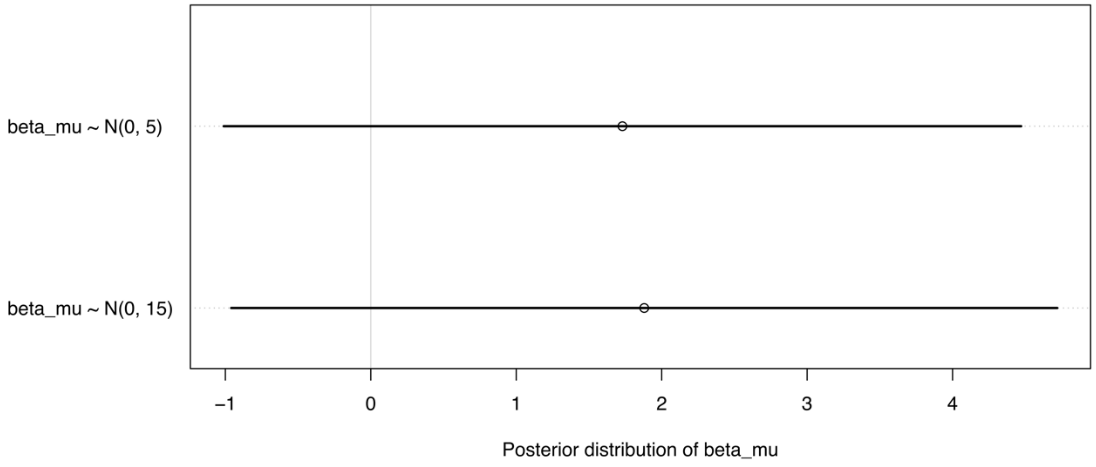

Figure S8: Testing the sensitivity of the posterior distribution to a weaker prior distribution for the parameter *beta_mu* (β_μ) for the Tasmanian data. The points and lines show the means and 95% credible intervals of the posterior distribution of the parameter β_μ obtained with each of the prior distributions indicated on the y-axis. (The y-axis shows the changes that were made to the model described in Table S4).

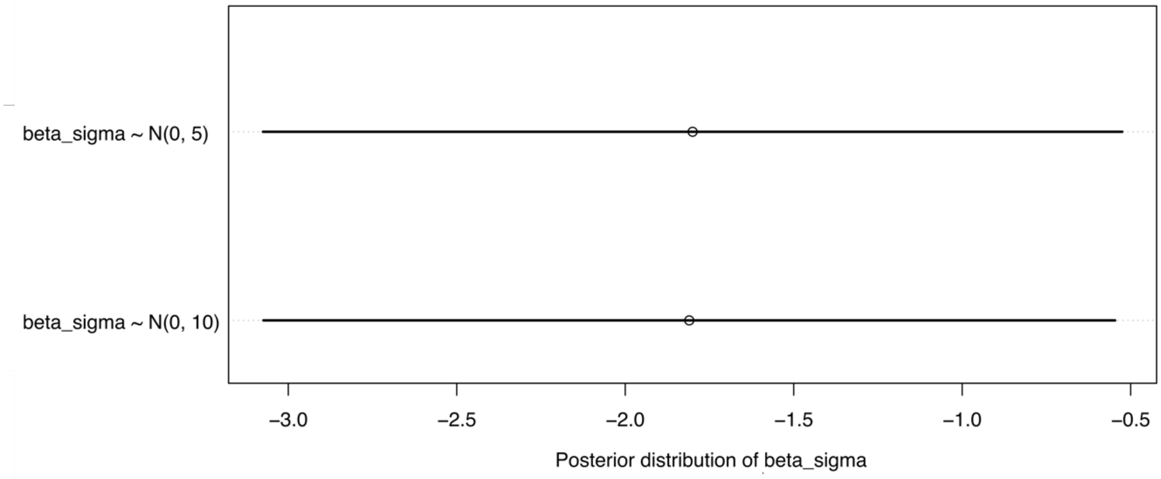

Figure S9: Testing the sensitivity of the posterior distribution to a weaker prior distribution for the parameter *beta_sigma* (β_σ) on the prior for the north-eastern New Zealand data. The points and lines show the means and 95% credible intervals of the posterior distribution of the parameter β_σ obtained with each of the prior distributions indicated on the y-axis. (The y-axis shows the changes that were made to the model described in Table S4).

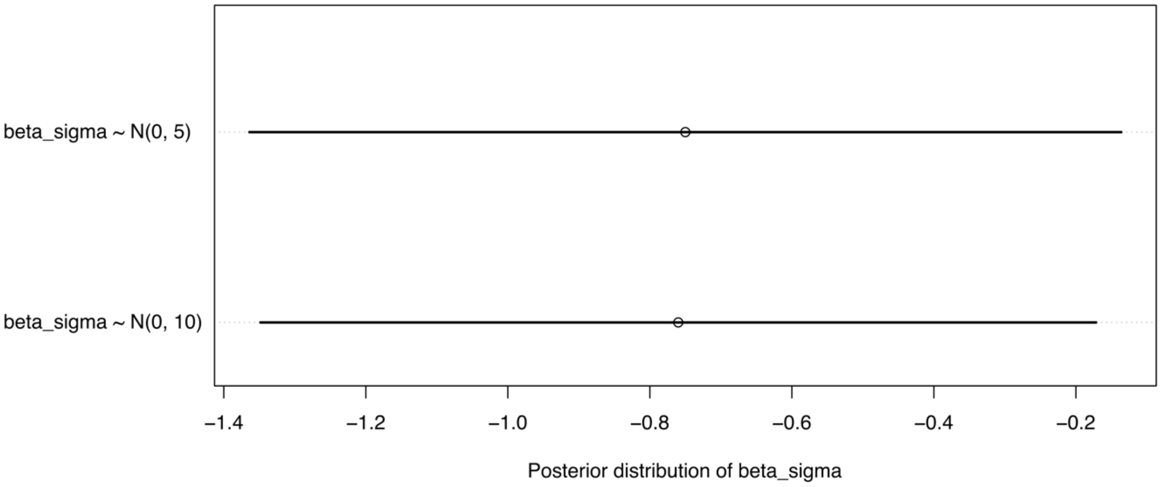

Figure S10: Testing the sensitivity of the posterior distribution to a weaker prior distribution for the parameter *beta_sigma* (β_σ) on the prior for the Tasmanian data. The points and lines show the means and 95% credible intervals of the posterior distribution of the parameter β_σ obtained with each of the prior distributions indicated on the y-axis. (The y-axis shows the changes that were made to the model described in Table S4).

3. Simulation study examining relationships between latitude and location means and standard deviations of urchin test diameters

3.1 Background for simulation study

The Long-spined sea urchin (*Centrostephanus rodgersii*) is found in both Tasmania and north-eastern New Zealand. In Tasmania, *C. rodgersii* extended its range poleward, which dramatically changed reef ecosystems and collapsed lobster and abalone fisheries. During the range extension, the more southerly populations of *C. rodgersii* had smaller mean test diameters (size) and younger mean age than more northerly populations (Ling et al. 2009).

It is currently unknown whether *C. rodgersii* is undergoing a similar range extension in north-eastern New Zealand. We measured test diameters from populations of *C. rodgersii,* distributed along a 7° latitudinal gradient from Spirits Bay (Kapowairua) to White Island (Whakaari), to look for a decline in mean test diameters and standard deviations of test diameters with increasing latitude (north to south) for *C. rodgersii* in New Zealand, in accordance with the pattern observed in Tasmania for this species.

We created a Bayesian model using the R package Rethinking (McElreath 2020) to estimate the location means and standard deviations of test diameters and regressed these estimates on latitude within a single model. We performed a simulation study to confirm that our model parameterization was appropriate for testing our hypotheses relating to relationships between location-specific summary statistics and latitude. The simulation study had three aims: (3.3) to test that that our model would recover the correct direction of a linear relationship (positive, negative, and neutral) for both the mean and standard deviation with latitude; (3.4) to assess the sensitivity of the model to different magnitudes of the mean and standard deviation regression parameters; and (3.5) to evaluate if we can accurately recover the model estimates for location means and standard deviations. In addition (3.6), we explored whether non-linear relationships between location-specific parameters and latitude affected the ability of the model to detect linear trends.

3.2 Summary of results

Overall, we found close agreement between the simulated location-specific parameters and the model estimates of these parameters across a range of realistic scenarios. Our model was conservative, slightly underestimating the strength of the relationship between latitude and the summary statistics when strong linear relationships were simulated. When a non-linear, relationship between latitude and the summary statistics was simulated, and a linear model was fit to these data, the relationship and the location estimates for the simulated mean test diameter were recovered, but the location estimates for the simulated standard deviations were not reliably recovered. Overall, our simulation study indicates that, if present, our model can reliably detect linear relationships between latitude and the location means and standard deviations of test diameters in *C. rodgersii* populations in north-eastern New Zealand.

3.3 Recovering linear relationships

For each scenario below we simulated two data sets with two different sample sizes: five locations (ending in "_5") and fifteen locations (ending in "_15"). Within each location, we simulated test diameter values for 30 individuals across all simulations.

Our nine simulated scenarios were:

sim_mn_vn_5/sim_mn_vn_15: no relationship between location means and latitude ("mn": bL = 0) and no relationship between location standard deviations and latitude ("vn": cL = 0)

sim_mi_vn_5/sim_mi_vn_15: positive relationship between location means and latitude ("mi": bL = 10) and no relationship between location standard deviations and latitude ("vn": cL = 0)

sim_md_vn_5/sim_md_vn_15: negative relationship between location means and latitude ("md": bL = -10) and no relationship between location standard deviations and latitude ("vn": cL = 0)

sim_mn_vi_5/sim_mn_vi_15: no relationship between location means and latitude ("mn": bL = 0) and positive relationship between location standard deviations and latitude ("vi": cL = 5)

sim_mi_vi_5/sim_mi_vi_15: positive relationship between location means and latitude ("mi": bL = 10) and positive relationship between location standard deviations and latitude ("vi": cL = 5)

sim_md_vi_5/sim_md_vi_15: negative relationship between location means and latitude ("md": bL = -10) and positive relationship between location standard deviations and latitude ("vi": cL = 5)

sim_mn_vd_5/sim_mn_vd_15: no relationship between location means and latitude ("mn": bL = 0) and negative relationship between location standard deviations and latitude ("vd": cL = -5)

sim_mi_vd_5/sim_mi_vd_15: positive relationship between location means and latitude ("mi": bL = 10) and negative relationship between location standard deviations and latitude ("vd": cL = -5)

sim_md_vd_5/sim_md_vd_15: negative relationship between location means and latitude ("md": bL = -10) and negative relationship between location standard deviations and latitude ("vd": cL = -5)

For each simulated dataset we then fit a model with the following parameters (for more information on the formal notation in brackets see section 2.1):

TD: Test diameter of urchins in mm (y_{ij}).

sL: Latitude standardised to a mean of 0 and standard deviation of 1 (x_i).

P: Urchin location (i).

mu: Mean test diameter at a location (μ_i).

mu_a: Global mean test diameter (α_μ).

bL: Slope of the relationship between standardised latitude and the location mean of test diameter (β_μ).

sigma_a and z_mu[P]: Error term for the mean test diameter of a location using non-centered parameterisation. The z_mu [P] allows the error to differ for each location ($z_{\mu,i}\tau_\mu$).

sigma: standard deviation of test diameters at a location (σ_i).

mu_c: Global standard deviation of test diameter (α_σ).

cL: Slope of the relationship between standardised latitude and the location standard deviation of test diameter (β_σ).

sigma_c and z_sigma[P]: Error term for the standard deviation of test diameters of a location using non-centered parameterisation. The z_sigma[P] allows the error to differ for each location ($z_{\sigma,i}\tau_\sigma$).

Data simulation and model function

The function below was written using the ulam function from the rethinking package.

```
data.sim_out <- function(pop, ind, lat, mu, sig){
  diam_m.v <- cbind(seq(mu[1], mu[2], length = pop), seq(sig[1], sig[2], length = pop))
  dat <- list(sL = rep(seq(lat[1], lat[2], length = pop), each = ind), TD = c(apply(diam_m.v, 1,
function(x) rnorm(ind, x[1], x[2]))), P = rep(seq(1, pop), each = ind))
  out <- ulam(
   alist(
    TD ~ dnorm(mu , sigma),
    mu <- mu_a + bL*sL + z_mu[P]*sigma_a,
    z_mu[P] ~ dnorm( 0, 1),
    sigma_a ~ dexp(2),
    mu_a ~ dnorm(90, 10),
    bL ~ dnorm( 0 , 5 ),
    sigma <- mu_c + cL*sL + z_sigma[P]*sigma_c,
    cL ~ dnorm( 0 , 5 ),
    mu_c ~ dnorm(15, 5),
```

```
    z_sigma[P] ~ dnorm( 0, 1),
    sigma_c ~ dexp(2)
  ), data= dat , chains=4, cores=1 , control=list(adapt_delta=0.99, max_treedepth = 15), log_lik
= TRUE, iter=3000, constraints=list( mu_c="lower=0", mu_a="lower=0"))
 out
}
```

Simulating data and generating model output for each of the nine scenarios for both 5 and 15 locations

```
sim_mn_vn_5 <- data.sim_out(pop = 5, ind = 30, lat = c(-1, 1), mu = c(90,90), sig = c(15,15))
sim_mi_vn_5 <- data.sim_out(pop = 5, ind = 30, lat = c(-1, 1), mu = c(80,100), sig = c(15,15))
sim_md_vn_5 <- data.sim_out(pop = 5, ind = 30, lat = c(-1, 1), mu = c(100,80), sig = c(15,15))
sim_mn_vi_5 <- data.sim_out(pop = 5, ind = 30, lat = c(-1, 1), mu = c(90,90), sig = c(10,20))
sim_mi_vi_5 <- data.sim_out(pop = 5, ind = 30, lat = c(-1, 1), mu = c(80,100), sig = c(10,20))
sim_md_vi_5 <- data.sim_out(pop = 5, ind = 30, lat = c(-1, 1), mu = c(100,80), sig = c(10,20))
sim_mn_vd_5 <- data.sim_out(pop = 5, ind = 30, lat = c(-1, 1), mu = c(90,90), sig = c(20,10))
sim_mi_vd_5 <- data.sim_out(pop = 5, ind = 30, lat = c(-1, 1), mu = c(80,100), sig = c(20,10))
sim_md_vd_5 <- data.sim_out(pop = 5, ind = 30, lat = c(-1, 1), mu = c(100,80), sig = c(20,10))

sim_mn_vn_15 <- data.sim_out(pop = 15, ind = 30, lat = c(-1, 1), mu = c(90,90), sig = c(15,15))
sim_mi_vn_15 <- data.sim_out(pop = 15, ind = 30, lat = c(-1, 1), mu = c(80,100), sig = c(15,15))
sim_md_vn_15 <- data.sim_out(pop = 15, ind = 30, lat = c(-1, 1), mu = c(100,80), sig = c(15,15))
sim_mn_vi_15 <- data.sim_out(pop = 15, ind = 30, lat = c(-1, 1), mu = c(90,90), sig = c(10,20))
sim_mi_vi_15 <- data.sim_out(pop = 15, ind = 30, lat = c(-1, 1), mu = c(80,100), sig = c(10,20))
sim_md_vi_15 <- data.sim_out(pop = 15, ind = 30, lat = c(-1, 1), mu = c(100,80), sig = c(10,20))
sim_mn_vd_15 <- data.sim_out(pop = 15, ind = 30, lat = c(-1, 1), mu = c(90,90), sig = c(20,10))
sim_mi_vd_15 <- data.sim_out(pop = 15, ind = 30, lat = c(-1, 1), mu = c(80,100), sig = c(20,10))
sim_md_vd_15 <- data.sim_out(pop = 15, ind = 30, lat = c(-1, 1), mu = c(100,80), sig = c(20,10))
```

Function to plot regression coefficients

The following function based of the coeftab_plot function plots the regression coefficients (± 95% highest posterior density interval [HPDI]) for the relationship between latitude and the location mean (bL) or location standard deviation (cL) in each scenario (rows) as well as the simulated bL an cL.

```
coeftab_plot_with_sim_estimates <- function (x, pars, sim.est, col.ci = "black", by.model =
FALSE, prob = 0.95, xlab = "Value", cex) {
 xse <- x@se
 x <- x@coefs
 if (!missing(pars)) {
  x <- x[pars, ]
  xse <- xse[pars, ]
```

```
    sim.est <- sim.est[pars, ]
  }
  if (by.model == FALSE) {
    xse <- t(xse)
    x <- t(x)
    sim.est <- t(sim.est)
  }
  z <- qnorm(1 - (1 - prob)/2)
  left <- x
  right <- x
  for (k in 1:nrow(x)) {
    for (m in 1:ncol(x)) {
      ci <- x[k, m] + c(-1, 1) * z * xse[k, m]
      left[k, m] <- ci[1]
      right[k, m] <- ci[2]
    }
  }
  llim <- min(left, na.rm = TRUE)
  rlim <- max(right, na.rm = TRUE)
  dotchart(x, xlab = xlab, xlim = c(llim, rlim), cex=cex)
  for (k in 1:nrow(x)) {
    for (m in 1:ncol(x)) {
      if (!is.na(left[k, m])) {
        kn <- nrow(x)
        ytop <- ncol(x) * (kn + 2) - 1
        ypos <- ytop - (m - 1) * (kn + 2) - (kn - k + 1)
        lines(c(left[k, m], right[k, m]), c(ypos, ypos), lwd = 2, col = col.ci)
        points(sim.est[k,m], ypos, cex = cex, pch = 1, col = "red")
      }
    }
  }
  abline(v = 0, lty = 1, col = col.alpha("black", 0.15))
}
```

Plotting the nine scenarios for both 5 and 15 locations

Plotting regression coefficients (± 95% HPDI) for the relationship between latitude and the location mean (bL) or location standard deviation (cL) for scenarios with different magnitudes of the regression coefficient (rows).

```
md <- -10
mn <- -0
mi <- 10
vd <- -5
```

```r
vn <- 0
vi <- 5

x_sim.est <- matrix(1:36,2,18)
x_sim.est[1,]<-rep(c(mn,mn,mi,mi,md,md), times = 3)
x_sim.est[2,]<-rep(c(vn,vi,vd), each = 6)
rownames(x_sim.est) <- paste(c("bL","cL"))

coeftab_plot_with_sim_estimates(coeftab(sim_mn_vn_5, sim_mn_vn_15, sim_mi_vn_5,
sim_mi_vn_15, sim_md_vn_5, sim_md_vn_15, sim_mn_vi_5, sim_mn_vi_15, sim_mi_vi_5,
sim_mi_vi_15, sim_md_vi_5, sim_md_vi_15, sim_mn_vd_5, sim_mn_vd_15, sim_mi_vd_5,
sim_mi_vd_15, sim_md_vd_5, sim_md_vd_15), pars = c("bL", "cL"), sim.est=x_sim.est, cex=0.5)
```

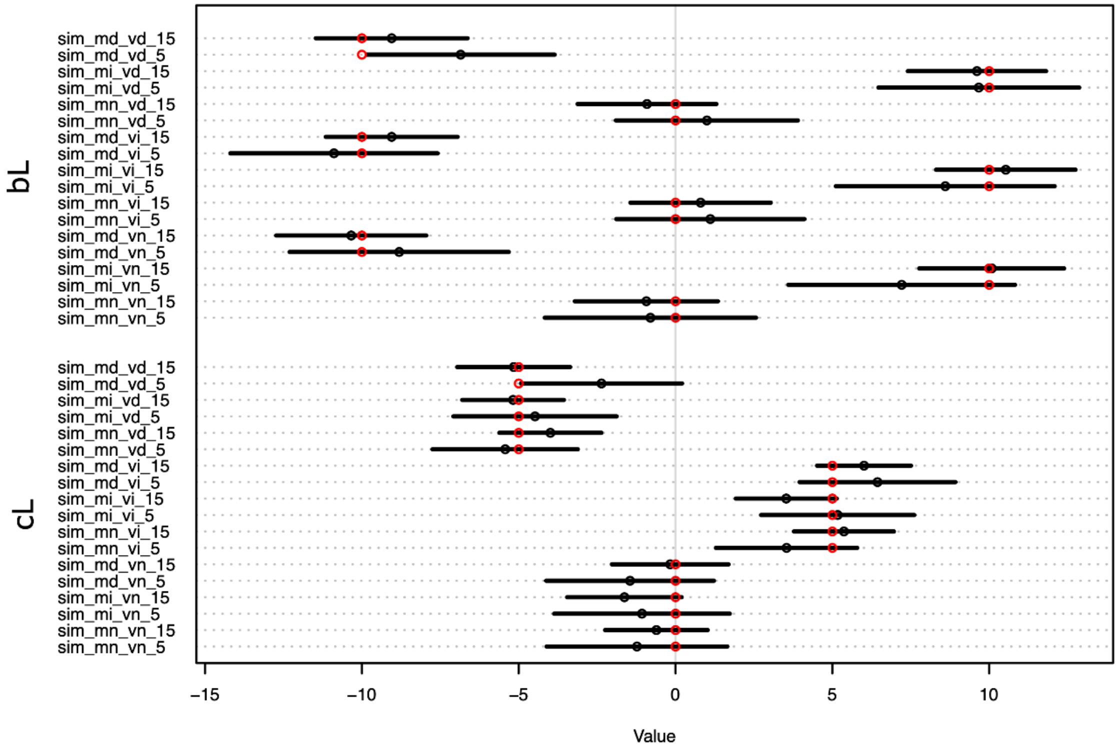

Figure S11: Plot of the regression coefficients bL (between latitude and the mean of test diameters of locations) and cL (between latitude and the variance of test diameters of locations). These were plotted for each of the nine simulated scenarios for both 15 location and 5 location datasets. The red circles are the simulated value for each of the parameters in each simulated scenario, the black circles are the posterior mean estimates and the lines are the 95% HPDI. Note: this is the same figure as Fig. 2 the main publication.

In summary, Figure S11 shows there was good agreement between the simulation parameters and the estimated coefficients and that the accuracy and precision of our estimated coefficients improve with greater sample sizes (i.e. comparing the accuracy of the 5 and 15 location datasets for the same scenario).

3.4 Testing different magnitudes of the regression parameters

Next, we explored if our model would be sensitive to differences in the magnitude of the regression parameters as well as differences in direction. Hence, we simulated six additional datasets under the following scenarios for both 5 (ending in _5) and 15 locations (ending in _15):

Changing the magnitude of bL:

sim_mi_low_vn_5/sim_mi_low_vn_15: low positive relationship between location means and increasing latitude (“mi_low”: bL = 5) and no relationship between location variances and latitude (“vn”: cL = 0)

sim_mi_med_vn_5/sim_mi_med_vn_15: medium positive relationship between location means and increasing latitude (“mi_med”: bL = 10) and no relationship between location variances and latitude (“vn”: cL = 0)

sim_mi_hig_vn_5/sim_mi_hig_vn_15: high positive relationship between location means and increasing latitude (“mi_hig”: bL = 20) and no relationship between location variances and latitude (“vn”: cL = 0)

Changing the magnitude of cL:

sim_mn_vi_low_5/sim_mn_vi_low_15: no relationship between location means and latitude (“mn”: bL = 0) and low positive relationship between location standard deviations and latitude (“vi_low”: cL = 2)

sim_mn_vi_med_5/sim_mn_vi_med_15: no relationship between location means and latitude (“mn”: bL = 0) and medium positive relationship between location standard deviations and latitude (“vi_med”: cL = 5)

sim_mn_vi_hig_5/sim_mn_vi_hig_15: no relationship between location means and latitude (“mn”: bL = 0) and high positive relationship between location standard deviations and latitude (“vi_hig”: cL = 10)

Simulating data and generating model output for each of the six scenarios for both 5 and 15 locations

```
sim_mi_low_vn_5 <- data.sim_out(pop = 5, ind = 30, lat = c(-1, 1), mu = c(85,95), sig = c(15,15))
sim_mi_med_vn_5 <- data.sim_out(pop = 5, ind = 30, lat = c(-1, 1), mu = c(80,100), sig =
c(15,15))
sim_mi_hig_vn_5 <- data.sim_out(pop = 5, ind = 30, lat = c(-1, 1), mu = c(70,110), sig = c(15,15))

sim_mn_vi_low_5 <- data.sim_out(pop = 5, ind = 30, lat = c(-1, 1), mu = c(90,90), sig = c(13,17))
sim_mn_vi_med_5 <- data.sim_out(pop = 5, ind = 30, lat = c(-1, 1), mu = c(90,90), sig =
c(10,20))
sim_mn_vi_hig_5 <- data.sim_out(pop = 5, ind = 30, lat = c(-1, 1), mu = c(90,90), sig = c(5,25))

sim_mi_low_vn_15 <- data.sim_out(pop = 15, ind = 30, lat = c(-1, 1), mu = c(85,95), sig =
c(15,15))
sim_mi_med_vn_15 <- data.sim_out(pop = 15, ind = 30, lat = c(-1, 1), mu = c(80,100), sig =
c(15,15))
sim_mi_hig_vn_15 <- data.sim_out(pop = 15, ind = 30, lat = c(-1, 1), mu = c(70,110), sig =
c(15,15))

sim_mn_vi_low_15 <- data.sim_out(pop = 15, ind = 30, lat = c(-1, 1), mu = c(90,90), sig =
c(13,17))
sim_mn_vi_med_15 <- data.sim_out(pop = 15, ind = 30, lat = c(-1, 1), mu = c(90,90), sig =
c(10,20))
sim_mn_vi_hig_15 <- data.sim_out(pop = 15, ind = 30, lat = c(-1, 1), mu = c(90,90), sig = c(5,25))
```

Plotting the six scenarios for both 5 and 15 locations

Plotting regression coefficients (± 95% HPDI) for the relationship between latitude and the location mean (bL) or location standard deviation (cL) for scenarios with different magnitudes of the regression coefficient (rows).

```
x_sim.est <- matrix(1:24,2,12)
x_sim.est[1,]<-c(0,0,0,0,0,0,5,5,10,10,20,20)
x_sim.est[2,]<-c(2,2,5,5,10,10,0,0,0,0,0,0)
rownames(x_sim.est) <- paste(c("bL","cL"))
colnames(x_sim.est) <- letters[1:12]

coeftab_plot_with_sim_estimates(coeftab(sim_mn_vi_low_5, sim_mn_vi_low_15,
sim_mn_vi_med_5, sim_mn_vi_med_15, sim_mn_vi_hig_5, sim_mn_vi_hig_15,
sim_mi_low_vn_5, sim_mi_low_vn_15, sim_mi_med_vn_5, sim_mi_med_vn_15,
sim_mi_hig_vn_5, sim_mi_hig_vn_15), pars = c("bL", "cL"), sim.est=x_sim.est, cex=0.5)
```

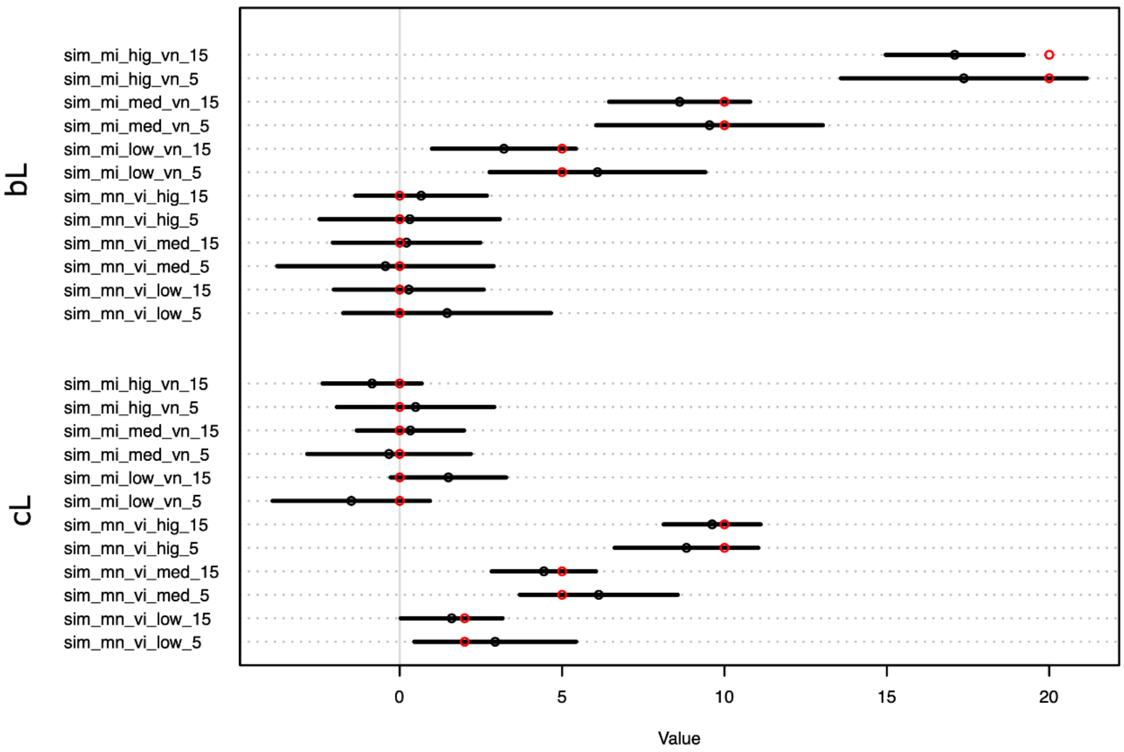

Figure S12: Plot of the regression coefficients bL (between latitude and the mean of test diameters of locations) and cL (between latitude and the variance of test diameters of locations). These were plotted for each of the 3 simulated scenarios for the change in the magnitude of bL and the change in the magnitude of cL for both 15 location and 5 location datasets. The red circles are the simulated values for each parameter in each simulated scenario, the black circles are the posterior mean estimates and the lines are the 95% HPDI.

In summary, Figure S12 shows there was good agreement between the simulation parameters and the estimated coefficients for all models except for the estimate of bL in model *sim_mi_hig_vn_15* suggesting that for steeper slopes the model estimates tend to underestimate the magnitude of the relationship. Nevertheless, although the *sim_mi_hig_vn_15* 95% HPDI of bL did not include the simulated bL it did have a greater magnitude of bL than *sim_mi_med_vn_15* so the estimates showed an increase in magnitude.

3.5 Recovering location means and location standard deviations

We examined if the estimates of the location means and location standard deviations were reliably recovered.

Functions to extract the location means and location standard deviation from the model output

```
all_model_samples<- function(modelname){
  p<-extract.samples(modelname)
  mu_out <- matrix(nrow= nrow(p$z), ncol = ncol(p$z))
  sigma_out <- matrix(nrow= nrow(p$z), ncol = ncol(p$z))
  for (i in 1:nrow(p$z)){
    mu_out[i,] <- p$mu_a[i] + p$bL[i]*unique(modelname@data$sL) +
p$z_mu[i,]*as.numeric(p$sigma_a[i])
    sigma_out[i,] <- p$mu_c[i] + p$cL[i]*unique(modelname@data$sL) +
p$z_sigma[i,]*as.numeric(p$sigma_c[i])
  }
  list(mu_out,sigma_out)
}


means_TD<-function(modelname){
  mu<-apply(all_model_samples(modelname)[[1]],2,mean)
  sd<-apply(all_model_samples(modelname)[[2]],2,mean)
  list(mu,sd)
}

model_credible_intervals_means<-function(modelname){
  samples_mu<-all_model_samples(modelname)[[1]]
  samples_sd<-all_model_samples(modelname)[[2]]
  ci_out_mu<-matrix(nrow=2,ncol=length(unique(modelname@data$P)))
  ci_out_sd<-matrix(nrow=2,ncol=length(unique(modelname@data$P)))
  for (i in 1:length(unique(modelname@data$P))){
    ci_out_mu[1,i]<-PI(samples_mu[,i], prob=0.95)[1]
   ci_out_mu[2,i]<-PI(samples_mu[,i], prob=0.95)[2]
   ci_out_sd[1,i]<-PI(samples_sd[,i], prob=0.95)[1]
   ci_out_sd[2,i]<-PI(samples_sd[,i], prob=0.95)[2]
  }
  list(ci_out_mu, ci_out_sd)
}
```

Plotting the estimated location means and location standard deviations for different directional relationships between the location means and latitude

Given that we had good agreement between the simulated parameters and the model regression parameters, we explored the accuracy of the model estimates of the location means and standard deviations.

First, we plotted the estimated location means and location standard deviations for simulated datasets with 15 populations but different relationships between the latitude and location means (positive, negative and none) and no relationship between latitude and locations standard deviations. The following models were plotted:

sim_mn_vn_15: no relationship between location means and latitude ("mn": bL = 0) and no relationship between location standard deviations and latitude ("vn": cL = 0)

sim_mi_vn_15: positive relationship between location means and latitude ("mi": bL = 10) and no relationship between location standard deviations and latitude ("vn": cL = 0)

sim_md_vn_15: negative relationship between location means and latitude ("md": bL = -10) and no relationship between location standard deviations and latitude ("vn": cL = 0)

Then, we plotted the estimated location means and locations standard deviations for simulated datasets with 15 populations but different relationships between the latitude and location standard deviations (positive, negative and none) and no relationship between latitude and locations means.

sim_mn_vn_15: no relationship between location means and latitude ("mn": bL = 0) and no relationship between location standard deviations and latitude ("vn": cL = 0)

sim_mn_vi_15: no relationship between location means and latitude ("mn": bL = 0) and positive relationship between location standard deviations and latitude ("vi": cL = 5)

sim_mn_vd_15: no relationship between location means and latitude ("mn": bL = 0) and negative relationship between location standard deviations and latitude ("vd": cL = -5)

```
plot_location_means_and_sd<-function(modelname, start, end, colour,
m_sd){points(unique(modelname@data$sL), means_TD(modelname)[[m_sd]], col=colour,
pch=16)
arrows(unique(modelname@data$sL),model_credible_intervals_means(modelname)[[m_sd]][
1,],y1=model_credible_intervals_means(modelname)[[m_sd]][2,],length = 0, col=colour)
points(seq(-1,1,length=length(unique(modelname@data$P))),seq(start,
end,length=length(unique(modelname@data$P))), col=colour, pch=1)
}
```

```
#m_sd=1 for location means and m_sd=2 for location standard deviations

par(mfrow = c(2, 2), mar = c(4,4,2,2))

plot(NULL, xlim=c(-1,1), ylim=c(75,105), xlab="latitude", ylab="test diameter", cex.axis=1.1,
cex.lab=1.1, main="a", cex.main=1.3)
plot_location_means_and_sd(modelname=sim_mn_vn_15, start=90, end=90, colour="blue",
m_sd=1)
plot_location_means_and_sd(modelname=sim_mi_vn_15, start=80, end=100,
colour="magenta", m_sd=1)
plot_location_means_and_sd(modelname=sim_md_vn_15, start=100, end=80,
colour="green", m_sd=1)

plot(NULL, xlim=c(-1,1), ylim=c(5,25), xlab="latitude", ylab="standard deviation", cex.axis=1.1,
cex.lab=1.1, main="b", cex.main=1.3)
plot_location_means_and_sd(modelname=sim_mn_vn_15, start=15, end=15, colour="blue",
m_sd=2)
plot_location_means_and_sd(modelname=sim_mi_vn_15, start=15, end=15,
colour="magenta", m_sd=2)
plot_location_means_and_sd(modelname=sim_md_vn_15, start=15, end=15, colour="green",
m_sd=2)

plot(NULL, xlim=c(-1,1), ylim=c(75,105), xlab="latitude", ylab="test diameter", cex.axis=1.1,
cex.lab=1.1, main="c", cex.main=1.3)
plot_location_means_and_sd(modelname=sim_mn_vn_15, start=90, end=90, colour="blue",
m_sd=1)
plot_location_means_and_sd(modelname=sim_mn_vi_15, start=90, end=90,
colour="magenta", m_sd=1)
plot_location_means_and_sd(modelname=sim_mn_vd_15, start=90, end=90, colour="green",
m_sd=1)

plot(NULL, xlim=c(-1,1), ylim=c(5,25), xlab="latitude", ylab="standard deviation", cex.axis=1.1,
cex.lab=1.1, main="d", cex.main=1.3)
plot_location_means_and_sd(modelname=sim_mn_vn_15, start=15, end=15, colour="blue",
m_sd=2)
plot_location_means_and_sd(modelname=sim_mn_vi_15, start=10, end=20,
colour="magenta", m_sd=2)
plot_location_means_and_sd(modelname=sim_mn_vd_15, start=20, end=10, colour="green",
m_sd=2)
```

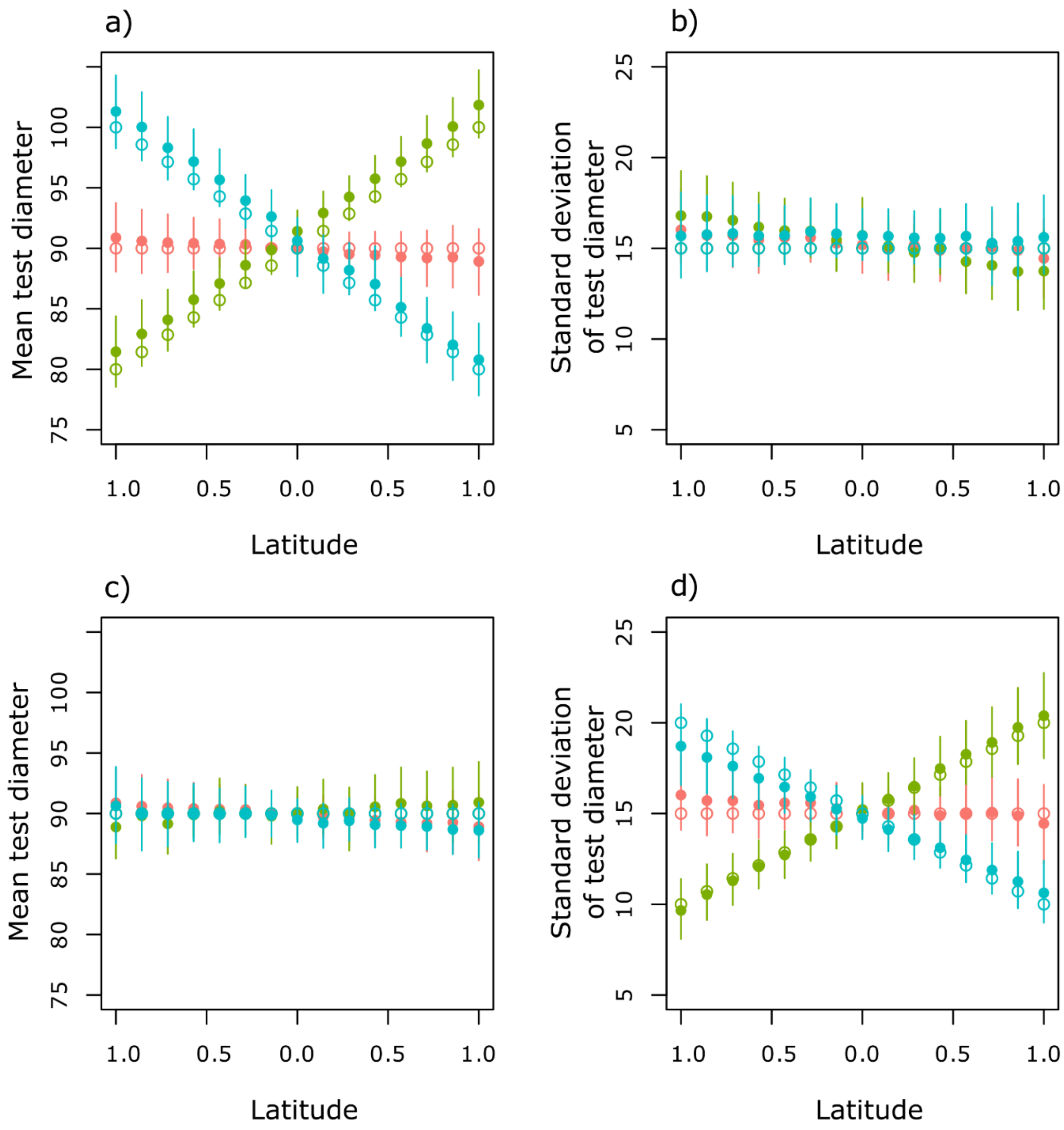

Figure S13: Plot of estimated and simulated location means in mm (a,c) and location standard deviations (b,d) for the following models: (a,b) *sim_mn_vn_15*: orange (no linear relationship between latitude and locations means and location standard deviations), *sim_mi_vn_15*: green (a positive relationship between locations means and latitude and no relationship between locations standard deviations and latitude), and *sim_md_vn_15*: blue (a negative relationship between locations means and latitude and no relationship between locations standard deviations and latitude); (c,d) *sim_mn_vn_15*: orange (no linear relationship between latitude and locations means and location standard deviations), *sim_mn_vi_15*: green (no relationship between locations means and latitude and a positive relationship between locations standard deviations and latitude), *sim_mn_vd_15*: blue (no relationship between locations means and latitude and a negative relationship between locations standard deviations and latitude). Closed symbols are the posterior mean and standard deviations for each location and open

symbols are the simulated location mean and standard deviations. The lines are the 95% HPDI. Note, that in panels b and c all models had the same simulated value, i.e. the simulated value is equal to zero.

The simulated locations means were within the 95% HPDI for the estimated location means for all 5 models. For both models *sim_mi_vn_15* and *sim_md_vn_15* the estimates of the location means were all slightly higher than was simulated (Fig. S13a).

The simulated location standard deviations were within the 95% HPDI for the estimated location standard deviations for all 5 models. Although within the 95% HPDI, there was a slight negative trend in location standard deviations for models *sim_mi_vn_15* and *sim_mn_vn_15* even when there was no simulated slope for location standard deviations. However, the simulated location standard deviations were still within the 95% HPDI (Fig. S11b). Similarly, although within the 95% HPDI, for model *sim_md_vn_15* the location standard deviations were all slightly higher than was simulated (Fig. S13b).

3.6 Testing the model with non-linear relationships between latitude and the location summary statistics

Lastly, we examined if non-linear patterns in the location means influenced our estimates for the linear relationships between latitude and the location means, and for no relationship between latitude and location standard deviations. These simulations used a convex relationship between latitude and either location means or standard deviations.

sim_mc_vn_5/sim_mc_vn_15: convex relationship between location means and latitude ("mc": bL = 0) and no relationship between location standard deviations and latitude ("vn": cL = 0)

sim_mn_vc_5/sim_mn_vc_15: no relationship between location means and latitude ("mn": bL = 0) and a convex relationship between location standard deviations and latitude ("vc": cL = 0)

Function to create non-linear relationships between latitude and location means or standard deviations using the ulam bayesian model from the rethinking package

```
data.sim_out.convex <- function(pop, ind, lat, mu, sig){
  diam_m.v <- cbind(mu, sig)
  dat <- list(sL = rep(seq(lat[1], lat[2], length = pop), each = ind), TD = c(apply(diam_m.v, 1,
function(x) rnorm(ind, x[1], (x[2])))), P = rep(seq(1, pop), each = ind))
  out <- ulam(
   alist(
    TD ~ dnorm(mu , sigma),
    mu <- mu_a + bL*sL + z_mu[P]*sigma_a,
    z_mu[P] ~ dnorm( 0, 1),
    sigma_a ~ dexp(2),
    mu_a ~ dnorm(90, 10),
    bL ~ dnorm( 0 , 5 ),
```

```
    sigma <- mu_c + cL*sL + z_sigma[P]*sigma_c,
    cL ~ dnorm( 0 , 5 ),
    mu_c ~ dnorm(15, 5),
    z_sigma[P] ~ dnorm( 0, 1),
    sigma_c ~ dexp(2)
  ), data= dat , chains=4, cores=1 , control=list(adapt_delta=0.99, max_treedepth = 15), log_lik
= TRUE, iter=3000, constraints=list( mu_c="lower=0", mu_a="lower=0"))
 out
}
```

Simulating data and generating model output for the two convex scenarios for both 5 and 15 locations

```
sim_mc_vn_5<-data.sim_out.convex(pop = 5, ind = 30, lat = c(-1, 1), mu = c(100,90,80,90,100),
sig = c(rep(15,5)))
sim_mn_vc_5<-data.sim_out.convex(pop = 5, ind = 30, lat = c(-1, 1), mu = c(rep(90,5)), sig =
c(20,15,10,15,20))

sim_mn_vc_15<-data.sim_out.convex(pop = 15, ind = 30, lat = c(-1, 1), mu = c(rep(90,15)), sig =
c(20,19,18,17,16,15,14,13,14,15,16,17,18,19,20))
sim_mc_vn_15<-data.sim_out.convex(pop = 15, ind = 30, lat = c(-1, 1), mu =
c(101,98,95,92,89,86,83,80,83,86,89,92,95,98,101), sig = c(rep(15,15)))
```

Plotting the the two convex scenarios for both 5 and 15 locations

Plotting regression coefficients (± HPDI) for the relationship between latitude and the location mean (bL) or location standard deviation (cL) for scenarios with a convex relationship between latitude and the location means.

```
x_sim.est <- matrix(1:8,2,4)
x_sim.est[1,]<-c(0,0,0,0)
x_sim.est[2,]<-c(0,0,0,0)
rownames(x_sim.est) <- paste(c("bL","cL"))

coeftab_plot_with_sim_estimates(coeftab(sim_mc_vn_5, sim_mc_vn_15, sim_mn_vc_5,
sim_mn_vc_15), pars = c("bL", "cL"), sim.est=x_sim.est, cex=1)
```

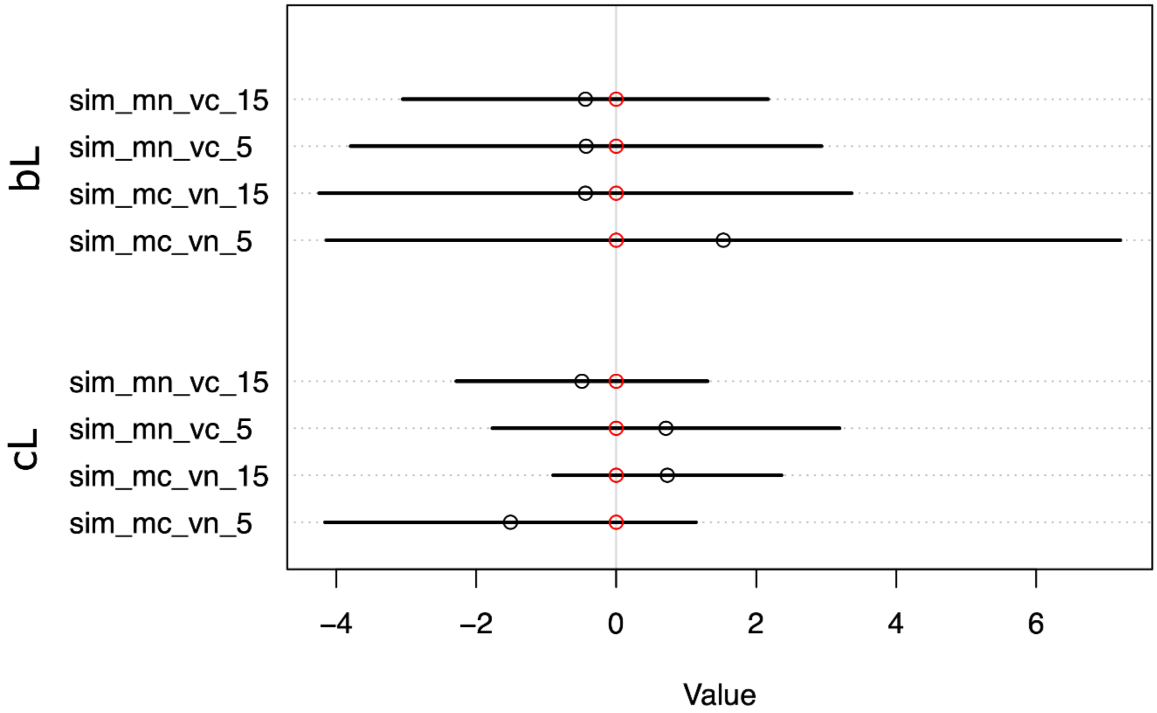

Figure S14: Plot of the regression coefficients bL (between latitude and the mean of test diameters of locations) and cL (between latitude and the variance of test diameters of locations). These were plotted for models: *sim_mc_vn_5/sim_mc_vn_15* (a convex relationship between the location means and latitude and no relationship between latitude and the location standard deviations) and *sim_mn_vc_5/sim_mn_vc_15* (no relationship between the location means and latitude and a convex relationship between latitude and the location standard deviations). The red circles are simulated values for each of the parameters in each of the simulated scenarios, the black circles are the posterior mean estimates and the lines are the 95% HPDI.

In summary, Figure S14 shows there was good agreement between the simulation parameters and the estimated coefficients. The convex relationship gave an estimate of 0 for bL in models *sim_mc_vn_5* and *sim_mc_vn_15* and an estimate of 0 for cL in models *sim_mn_vc_5* and *sim_mn_vc_15* as was expected.

Plotting the estimated location means and location standard deviations for a convex relationship between the location means and latitude but no relationship between latitude and the location standard deviations.

```
plot_location_means_and_sd_2<-function(modelname, seq, colour,
m_sd){points(unique(modelname@data$sL), means_TD(modelname)[[m_sd]], col=colour,
pch=16)
arrows(unique(modelname@data$sL),model_credible_intervals_means(modelname)[[m_sd]][
1,],y1=model_credible_intervals_means(modelname)[[m_sd]][2,],length = 0, col=colour)
points(seq(-1,1,length=length(unique(modelname@data$P))),seq, col=colour, pch=1)
}

#m_sd=1 for location means and m_sd=2 for location standard deviations

convex.means_5<-c(100,90,80,90,100)
convex.means_15<-c(101,98,95,92,89,86,83,80,83,86,89,92,95,98,101)
convex.sd_5<-c(20,15,10,15,20)
convex.sd_15<-c(20,19,18,17,16,15,14,13,14,15,16,17,18,19,20)

par(mfrow = c(2, 2), mar = c(4,4,2,2))

plot(NULL, xlim=c(-1,1), ylim=c(75,105), xlab="latitude", ylab="test diameter", cex.axis=1.1,
cex.lab=1.1, main="a", cex.main=1.3)
plot_location_means_and_sd_2(modelname=sim_mc_vn_5, seq=convex.means_5,
colour="purple", m_sd=1)
plot_location_means_and_sd(modelname=sim_mn_vc_5, start=90, end=90,
colour="#e34234", m_sd=1)

plot(NULL, xlim=c(-1,1), ylim=c(5,25), xlab="latitude", ylab="standard deviation", cex.axis=1.1,
cex.lab=1.1, main="b", cex.main=1.3)
plot_location_means_and_sd(modelname=sim_mc_vn_5, start=15, end=15, colour="purple",
m_sd=2)
plot_location_means_and_sd_2(modelname=sim_mn_vc_5, seq=convex.sd_5,
colour="#e34234", m_sd=2)

plot(NULL, xlim=c(-1,1), ylim=c(75,105), xlab="latitude", ylab="test diameter", cex.axis=1.1,
cex.lab=1.1, main="a", cex.main=1.3)
plot_location_means_and_sd_2(modelname=sim_mc_vn_15, seq=convex.means_15,
colour="purple", m_sd=1)
plot_location_means_and_sd(modelname=sim_mn_vc_15, start=90, end=90,
colour="#e34234", m_sd=1)

plot(NULL, xlim=c(-1,1), ylim=c(5,25), xlab="latitude", ylab="standard deviation", cex.axis=1.1,
cex.lab=1.1, main="b", cex.main=1.3)
```

```
plot_location_means_and_sd(modelname=sim_mc_vn_15, start=15, end=15, colour="purple", m_sd=2)
plot_location_means_and_sd_2(modelname=sim_mn_vc_15, seq=convex.sd_15, colour="#e34234", m_sd=2)
```

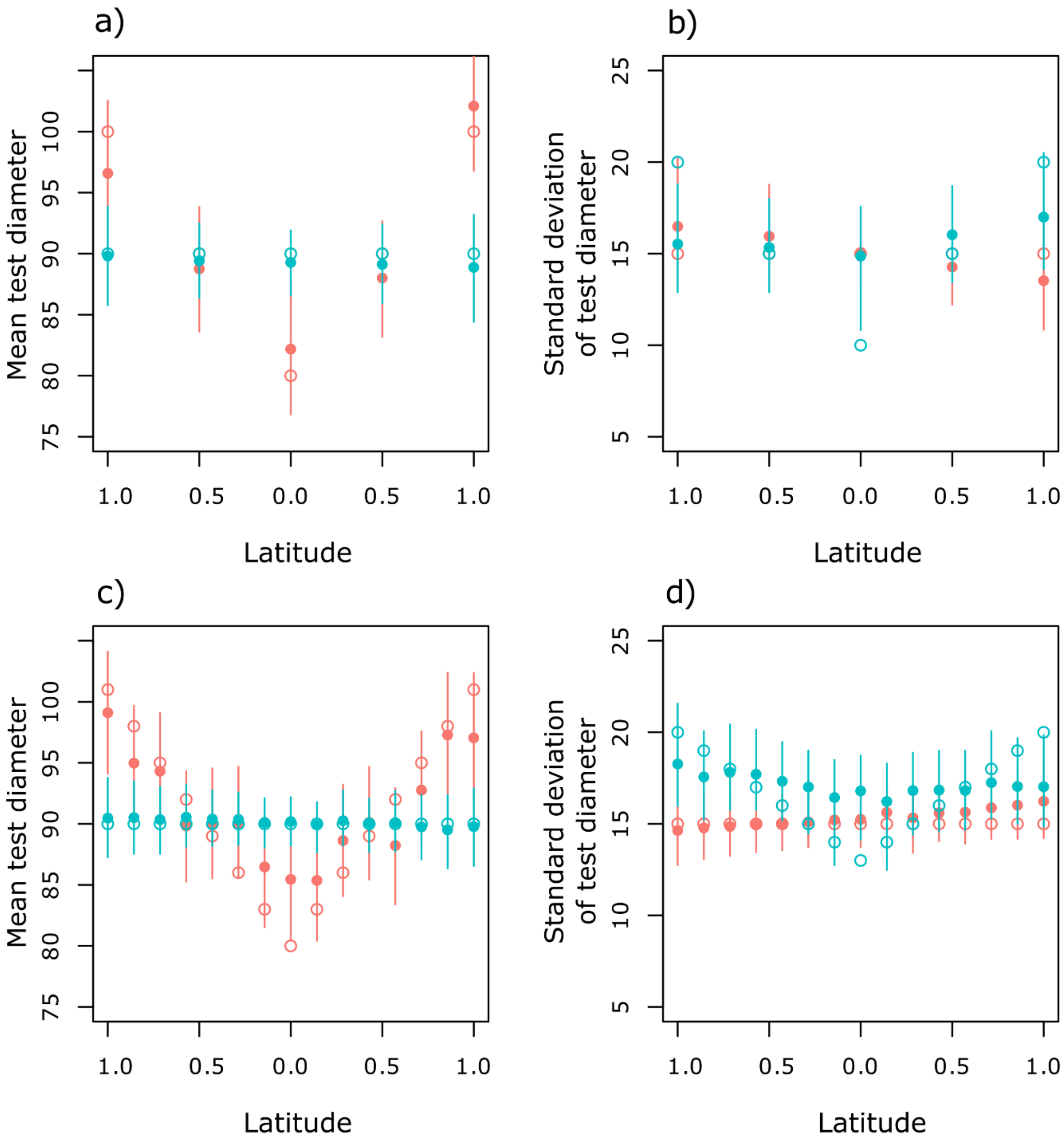

Figure S15: Plotting the estimated and simulated location means in mm (a,c) and location standard deviations (b,d) for models: *sim_mc_vn_5* (orange; a,b)/*sim_mc_vn_15* (orange; c,d) (convex relationship between the location means and latitude and no relationship between latitude and the location standard deviations) and *sim_mn_vc_5* (blue; a,b)/*sim_mn_vc_15* (blue; c,d) (no relationship

between the location means and latitude and a convex relationship between latitude and the location standard deviations). Closed symbols are the posterior mean and standard deviations for each location from the model and open symbols are the simulated location mean and standard deviations. The lines are the 95% HPDI.

For both the 15 location and 5 location simulations, the convex pattern of the location means and latitude was recovered (Fig. S15a,c). However, the extent of the convex pattern was less extreme for the estimated location means than the simulated location means. For model *sim_mc_vn_15*, the lowest simulated location mean at latitude 0 was below the 95% HPDI (Fig. S15c). The rest of the location means had a simulated location mean within the estimated 95% HPDI.

For both the 15 location and 5 location simulations, the convex pattern between the location standard deviations and latitude was not recovered (Fig. S15b,d). For model *sim_mn_vc_5*, the simulated location standard deviation at latitude -1 was above the 95% HPDI and the simulated location standard deviation at latitude 0 was below the 95% HPDI. Therefore, the convex pattern was not recovered and location standard deviation estimates were not accurate. For model *sim_mc_vn_*5, the slope for the location standard deviations was negative when there was no simulated slope, but the simulated location standard deviations were within the estimated HDPI errors. Model *sim_mc_vn_5* gave a positive slope for the location standard deviations when there was no simulated slope, but the simulated location standard deviations were within the estimated 95% HPDI. For model *sim_mn_vc_15*, the convex pattern for the location standard deviations was partially recovered. Although the points did not follow the simulated pattern, all the location standard deviations were within the 95% HPDIs except the location at latitude 1 for which the simulated location standard deviation was above the estimated 95% HPDI.

3.7 Supplementary tables: Outputs from models

These tables show the estimates for each of the parameters in the model: bL, cL, sigma_a, sigma_c, mu_a, mu_c, z_mu[1] to z_mu[5], and z_sigma[1] to z_sigma[5] (for 5 populations) or z_mu[1] to z_mu[15] and z_sigma[1] to z_sigma[15] (for 15 populations).

The estimates for the following 9 scenarios are shown for both 5 and 15 populations:

sim_mn_vn_5/sim_mn_vn_15: no relationship between location means and latitude ("mn": bL = 0) and no relationship between location standard deviations and latitude ("vn": cL = 0)

sim_mi_vn_5/sim_mi_vn_15: positive relationship between location means and latitude ("mi": bL = 10) and no relationship between location standard deviations and latitude ("vn": cL = 0)

sim_md_vn_5/sim_md_vn_15: negative relationship between location means and latitude ("md": bL = -10) and no relationship between location standard deviations and latitude ("vn": cL = 0)

sim_mn_vi_5/sim_mn_vi_15: no relationship between location means and latitude ("mn": bL = 0) and positive relationship between location standard deviations and latitude ("vi": cL = 5)

sim_mi_vi_5/sim_mi_vi_15: positive relationship between location means and latitude ("mi": bL = 10) and positive relationship between location standard deviations and latitude ("vi": cL = 5)

sim_md_vi_5/sim_md_vi_15: negative relationship between location means and latitude ("md": bL = -10) and positive relationship between location standard deviations and latitude ("vi": cL = 5)

sim_mn_vd_5/sim_mn_vd_15: no relationship between location means and latitude ("mn": bL = 0) and negative relationship between location standard deviations and latitude ("vd": cL = -5)

sim_mi_vd_5/sim_mi_vd_15: positive relationship between location means and latitude ("mi": bL = 10) and negative relationship between location standard deviations and latitude ("vd": cL = -5)

sim_md_vd_5/sim_md_vd_15: negative relationship between location means and latitude ("md": bL = -10) and negative relationship between location standard deviations and latitude ("vd": cL = -5)

```r
coeftab(sim_mn_vn_5, sim_mn_vn_15, sim_mi_vn_5, sim_mi_vn_15, sim_md_vn_5, sim_md_vn_15, sim_mn_vi_5, sim_mn_vi_15,
sim_mi_vi_5, sim_mi_vi_15, sim_md_vi_5, sim_md_vi_15, sim_mn_vd_5, sim_mn_vd_15, sim_mi_vd_5, sim_mi_vd_15,
sim_md_vd_5, sim_md_vd_15)

##            sim_mn_vn_5 sim_mn_vn_15 sim_mi_vn_5 sim_mi_vn_15 sim_md_vn_5 sim_md_vn_15 sim_mn_vi_5 sim_mn_vi_15
sim_mi_vi_5 sim_mi_vi_15
## z_mu[1]       0.01        0.08        -0.27        0.10        0.03        -0.05        -0.02        -0.45        -0.25        0.13
## z_mu[2]       0.01        -0.12        0.10        0.13        0.03        0.13        0.04        0.37        0.18        0.00
## z_mu[3]       -0.08        -0.11        0.30        -0.19        0.00        -0.08        -0.05        -0.39        -0.10        -0.05
## z_mu[4]       0.05        -0.01        -0.13        0.07        0.03        0.24        0.08        0.20        0.31        0.25
## z_mu[5]       -0.01        0.10        -0.01        -0.04        -0.11        0.19        0.00        0.39        -0.13        -0.53
## sigma_a       0.45        0.42        0.57        0.46        0.45        0.62        0.43        0.67        0.58        0.78
## mu_a          89.29        89.88        89.82        91.46        91.72        91.02        90.24        90.12        89.29        89.54
## bL            -0.80        -0.93        7.21        10.08        -8.81        -10.34        1.11        0.80        8.60        10.53
## cL            -1.23        -0.61        -1.07        -1.63        -1.45        -0.17        3.54        5.37        5.17        3.53
## mu_c          16.08        15.26        16.26        15.34        15.70        15.65        13.88        15.10        16.00        14.54
## z_sigma[1]    -0.12        0.21        0.01        -0.20        -0.12        -0.19        0.07        -0.13        0.08        -0.12
## z_sigma[2]    0.05        -0.10        -0.11        0.01        0.25        -0.07        -0.33        0.07        -0.16        0.04
## z_sigma[3]    0.12        0.02        0.16        0.05        -0.19        0.06        0.05        0.07        -0.07        0.10
## z_sigma[4]    0.25        -0.22        0.11        -0.13        0.06        -0.08        0.28        0.05        0.11        -0.03
## z_sigma[5]    -0.25        0.08        -0.14        -0.09        -0.03        -0.03        -0.12        -0.14        0.04        -0.06
## sigma_c       0.50        0.44        0.45        0.49        0.47        0.42        0.49        0.35        0.44        0.33
## z_mu[6]       NA        0.25        NA        0.03        NA        -0.04        NA        0.16        NA        0.26
## z_mu[7]       NA        -0.02        NA        -0.19        NA        0.12        NA        -0.21        NA        0.17
## z_mu[8]       NA        0.05        NA        -0.06        NA        -0.37        NA        -0.01        NA        -0.56
## z_mu[9]       NA        -0.05        NA        0.02        NA        -0.39        NA        0.14        NA        -0.09
## z_mu[10]       NA        -0.14        NA        -0.12        NA        0.12        NA        -0.41        NA        -0.35
## z_mu[11]       NA        -0.06        NA        -0.04        NA        0.43        NA        0.09        NA        0.08
## z_mu[12]       NA        -0.09        NA        -0.08        NA        0.04        NA        0.24        NA        0.49
## z_mu[13]       NA        -0.04        NA        -0.01        NA        -0.24        NA        -0.06        NA        -0.22
## z_mu[14]       NA        0.23        NA        -0.05        NA        -0.12        NA        -0.12        NA        0.31
```

```
## z_mu[15]      NA      -0.05       NA      0.39       NA      0.12       NA      0.00       NA      0.12
## z_sigma[6]    NA       0.21       NA      0.19       NA      0.31       NA      0.01       NA      0.02
## z_sigma[7]    NA      -0.09       NA     -0.13       NA      0.17       NA     -0.03       NA      0.08
## z_sigma[8]    NA      -0.10       NA      0.50       NA      0.04       NA      0.20       NA      0.01
## z_sigma[9]    NA      -0.24       NA      0.02       NA      0.05       NA     -0.11       NA     -0.09
## z_sigma[10]   NA       0.12       NA     -0.15       NA     -0.03       NA     -0.20       NA     -0.22
## z_sigma[11]   NA      -0.16       NA      0.45       NA     -0.04       NA      0.14       NA      0.13
## z_sigma[12]   NA       0.03       NA     -0.17       NA      0.16       NA      0.17       NA      0.19
## z_sigma[13]   NA       0.37       NA     -0.15       NA     -0.34       NA     -0.02       NA     -0.02
## z_sigma[14]   NA       0.19       NA     -0.28       NA     -0.15       NA      0.08       NA      0.09
## z_sigma[15]   NA      -0.28       NA      0.03       NA      0.17       NA     -0.13       NA     -0.12
## nobs         150      450      150      450      150      450      150      450      150      450
##         sim_md_vi_5 sim_md_vi_15 sim_mn_vd_5 sim_mn_vd_15 sim_mi_vd_5 sim_mi_vd_15 sim_md_vd_5 sim_md_vd_15
## z_mu[1]       0.10     0.04    -0.04     0.22    -0.14    -0.09     0.01     0.35
## z_mu[2]      -0.03    -0.25     0.10    -0.29     0.01    -0.09    -0.05    -0.21
## z_mu[3]       0.00     0.11    -0.01    -0.14     0.04     0.14     0.23     0.03
## z_mu[4]       0.09     0.25    -0.06     0.00     0.22    -0.06    -0.17     0.40
## z_mu[5]      -0.16    -0.04     0.04    -0.03    -0.09    -0.12    -0.08     0.09
## sigma_a       0.47     0.41     0.43     0.47     0.49     0.39     0.49     0.97
## mu_a         91.61    89.89    90.77    89.53    90.72    90.08    89.42    90.16
## bL          -10.89    -9.05     1.00    -0.91     9.67     9.61    -6.85    -9.05
## cL            6.44     6.01    -5.43    -3.99    -4.48    -5.18    -2.36    -5.16
## mu_c         15.82    14.55    13.63    14.72    14.87    15.10    14.12    15.23
## z_sigma[1]   -0.02    -0.08     0.15     0.00     0.05    -0.12    -0.15    -0.08
## z_sigma[2]   -0.07     0.12    -0.30    -0.10    -0.26     0.10     0.16     0.14
## z_sigma[3]    0.08     0.07     0.23     0.08     0.42     0.32     0.10    -0.50
## z_sigma[4]    0.00     0.08     0.02    -0.11    -0.03    -0.05     0.11    -0.12
## z_sigma[5]    0.02    -0.21    -0.10     0.05    -0.18     0.03    -0.17     0.32
## sigma_c       0.41     0.33     0.50     0.32     0.54     0.53     0.45     0.67
## z_mu[6]        NA      0.25       NA     0.26       NA     0.05       NA    -0.34
## z_mu[7]        NA     -0.12       NA     0.23       NA     0.11       NA    -0.06
```

## z_mu[8]	NA	-0.03	NA	-0.06	NA	-0.05	NA	0.02
## z_mu[9]	NA	-0.04	NA	-0.23	NA	-0.05	NA	-0.14
## z_mu[10]	NA	-0.06	NA	0.16	NA	0.10	NA	0.10
## z_mu[11]	NA	-0.06	NA	-0.09	NA	0.04	NA	0.03
## z_mu[12]	NA	0.13	NA	-0.01	NA	0.03	NA	-0.67
## z_mu[13]	NA	-0.15	NA	0.07	NA	0.15	NA	0.67
## z_mu[14]	NA	-0.05	NA	-0.07	NA	-0.04	NA	-0.71
## z_mu[15]	NA	0.04	NA	-0.01	NA	-0.11	NA	0.47
## z_sigma[6]	NA	-0.09	NA	0.12	NA	-0.05	NA	0.54
## z_sigma[7]	NA	0.01	NA	-0.08	NA	0.34	NA	0.26
## z_sigma[8]	NA	0.09	NA	0.03	NA	-0.42	NA	-0.42
## z_sigma[9]	NA	0.01	NA	-0.06	NA	-0.27	NA	0.08
## z_sigma[10]	NA	-0.05	NA	0.07	NA	-0.27	NA	0.14
## z_sigma[11]	NA	-0.04	NA	0.21	NA	0.33	NA	-0.12
## z_sigma[12]	NA	0.08	NA	0.02	NA	-0.40	NA	0.21
## z_sigma[13]	NA	-0.23	NA	0.05	NA	0.24	NA	-0.56
## z_sigma[14]	NA	0.15	NA	-0.07	NA	-0.03	NA	-0.01
## z_sigma[15]	NA	0.12	NA	-0.18	NA	0.21	NA	0.11
## nobs	150	450	150	450	150	450	150	450

Table S5: Nine different scenarios.

The estimates for the following 6 scenarios are shown for both 5 and 15 populations:

Change in bL magnitude:

sim_mi_low_vn_5/sim_mi_low_vn_15: low positive relationship between location means and increasing latitude (“mi_low”: bL = 5) and no relationship between location variances and latitude (“vn”: cL = 0)

sim_mi_med_vn_5/sim_mi_med_vn_15: medium positive relationship between location means and increasing latitude (“mi_med”: bL = 10) and no relationship between location variances and latitude (“vn”: cL = 0)

sim_mi_hig_vn_5/sim_mi_hig_vn_15: high positive relationship between location means and increasing latitude (“mi_hig”: bL = 20) and no relationship between location variances and latitude (“vn”: cL = 0)

Change in cL magnitude:

sim_mn_vi_low_5/sim_mn_vi_low_15: no relationship between location means and latitude (“mn”: bL = 0) and low positive relationship between location standard deviations and latitude (“vi_low”: cL = 2)

sim_mn_vi_med_5/sim_mn_vi_med_15: no relationship between location means and latitude (“mn”: bL = 0) and medium positive relationship between location standard deviations and latitude (“vi_med”: cL = 5)

sim_mn_vi_hig_5/sim_mn_vi_hig_15: no relationship between location means and latitude (“mn”: bL = 0) and high positive relationship between location standard deviations and latitude (“vi_hig”: cL = 10)

```r
coeftab(sim_mn_vi_low_5, sim_mn_vi_low_15, sim_mn_vi_med_5, sim_mn_vi_med_15, sim_mn_vi_hig_5, sim_mn_vi_hig_15,
sim_mi_low_vn_5, sim_mi_low_vn_15, sim_mi_med_vn_5, sim_mi_med_vn_15, sim_mi_hig_vn_5, sim_mi_hig_vn_15)

##          sim_mn_vi_low_5 sim_mn_vi_low_15 sim_mn_vi_med_5 sim_mn_vi_med_15 sim_mn_vi_hig_5 sim_mn_vi_hig_15
sim_mi_low_vn_5
## z_mu[1]      -0.01   -0.15   -0.02    0.07    0.05    0.18   -0.05
## z_mu[2]       0.06    0.09   -0.01    0.00   -0.13    0.01    0.00
## z_mu[3]      -0.17   -0.03    0.05   -0.20    0.06   -0.14    0.05
## z_mu[4]       0.17   -0.08   -0.06   -0.63    0.11   -0.21   -0.11
## z_mu[5]      -0.03    0.09    0.03    0.36   -0.08   -0.02    0.12
## sigma_a       0.48    0.67    0.44    0.79    0.44    0.39    0.45
## mu_a         91.51   90.17   92.13   89.45   88.52   89.34   90.25
## bL            1.46    0.28   -0.44    0.21    0.31    0.66    6.09
## cL            2.94    1.60    6.13    4.44    8.83    9.62   -1.49
## mu_c         14.81   14.77   15.79   14.84   13.82   15.37   15.25
## z_sigma[1]    0.00    0.19   -0.03    0.20   -0.27    0.04    0.02
## z_sigma[2]    0.05   -0.17    0.13   -0.29    0.33   -0.14    0.10
## z_sigma[3]   -0.28    0.02   -0.11    0.04   -0.21   -0.12   -0.07
## z_sigma[4]    0.27    0.01   -0.15   -0.24    0.09    0.05   -0.09
## z_sigma[5]   -0.07   -0.13    0.18    0.02    0.02    0.06    0.08
## sigma_c       0.49    0.33    0.46    0.42    0.49    0.31    0.43
## z_mu[6]         NA   -0.40      NA    0.16      NA    0.11      NA
## z_mu[7]         NA    0.08      NA    0.43      NA    0.19      NA
## z_mu[8]         NA    0.29      NA    0.20      NA   -0.15      NA
## z_mu[9]         NA    0.10      NA    0.11      NA    0.21      NA
## z_mu[10]        NA   -0.36      NA   -0.12      NA   -0.09      NA
## z_mu[11]        NA    0.54      NA    0.10      NA   -0.03      NA
## z_mu[12]        NA    0.26      NA   -0.33      NA   -0.05      NA
## z_mu[13]        NA   -0.13      NA   -0.44      NA   -0.09      NA
## z_mu[14]        NA   -0.14      NA   -0.12      NA    0.10      NA
## z_mu[15]        NA   -0.25      NA    0.43      NA    0.01      NA
```

```
## z_sigma[6]     NA          0.05            NA          0.28            NA          0.06            NA
## z_sigma[7]     NA         -0.08            NA         -0.09            NA          0.21            NA
## z_sigma[8]     NA          0.06            NA         -0.24            NA         -0.06            NA
## z_sigma[9]     NA         -0.09            NA         -0.04            NA          0.03            NA
## z_sigma[10]    NA          0.03            NA          0.34            NA         -0.10            NA
## z_sigma[11]    NA          0.08            NA          0.11            NA         -0.15            NA
## z_sigma[12]    NA         -0.15            NA          0.02            NA         -0.04            NA
## z_sigma[13]    NA          0.10            NA          0.01            NA          0.07            NA
## z_sigma[14]    NA         -0.02            NA         -0.06            NA         -0.09            NA
## z_sigma[15]    NA          0.07            NA         -0.11            NA          0.20            NA
## nobs          150          450           150          450           150          450           150
##        sim_mi_low_vn_15 sim_mi_med_vn_5 sim_mi_med_vn_15 sim_mi_hig_vn_5 sim_mi_hig_vn_15
## z_mu[1]       0.06          -0.16          -0.08          -0.56          0.12
## z_mu[2]       0.09           0.07          -0.10           0.18          0.02
## z_mu[3]       0.02          -0.06          -0.11           0.41         -0.16
## z_mu[4]      -0.02           0.13           0.13          -0.24         -0.17
## z_mu[5]      -0.24           0.00          -0.08           0.21          0.01
## sigma_a       0.42           0.48           0.45           0.82          0.40
## mu_a         90.02          90.59          90.03          88.96         90.49
## bL            3.21           9.54           8.62          17.37         17.09
## cL            1.50          -0.33           0.33           0.49         -0.85
## mu_c         15.14          15.90          14.96          15.64         14.10
## z_sigma[1]   -0.81           0.41          -0.07           0.21         -0.04
## z_sigma[2]    0.39          -0.43           0.02          -0.21         -0.06
## z_sigma[3]    0.51           0.03           0.11          -0.08          0.25
## z_sigma[4]    0.12          -0.38           0.08           0.00         -0.03
## z_sigma[5]    0.13           0.38          -0.10           0.09         -0.09
## sigma_c       0.80           0.76           0.38           0.47          0.33
## z_mu[6]      -0.14             NA           0.00             NA         -0.12
## z_mu[7]       0.08             NA           0.15             NA         -0.03
## z_mu[8]      -0.17             NA           0.24             NA          0.13
```

## z_mu[9]	0.23	NA	-0.01	NA	0.15
## z_mu[10]	0.11	NA	-0.03	NA	-0.08
## z_mu[11]	-0.05	NA	-0.18	NA	-0.14
## z_mu[12]	0.10	NA	0.04	NA	0.07
## z_mu[13]	0.02	NA	0.17	NA	-0.02
## z_mu[14]	0.02	NA	0.17	NA	0.08
## z_mu[15]	-0.08	NA	-0.22	NA	0.16
## z_sigma[6]	-0.11	NA	0.14	NA	0.08
## z_sigma[7]	-0.17	NA	-0.04	NA	-0.11
## z_sigma[8]	0.21	NA	-0.28	NA	-0.06
## z_sigma[9]	-0.47	NA	-0.06	NA	0.08
## z_sigma[10]	-0.25	NA	-0.06	NA	-0.09
## z_sigma[11]	-0.13	NA	0.12	NA	-0.11
## z_sigma[12]	0.39	NA	-0.08	NA	-0.01
## z_sigma[13]	0.37	NA	0.29	NA	0.27
## z_sigma[14]	0.15	NA	0.14	NA	-0.07
## z_sigma[15]	-0.39	NA	-0.21	NA	-0.04
## nobs	450	150	450	150	450

Table S6: Testing different magnitudes of the regression parameters.

The estimates for the following model is shown for 5 populations.

sim_mc_vn_5: convex relationship between location means and latitude ("mc": bL = 0) and no relationship between location standard deviations and latitude ("vn": cL = 0)

```
coeftab(sim_mc_vn_5)

##        sim_mc_vn_5
## z_mu[1]     1.44
## z_mu[2]    -0.42
## z_mu[3]       -2
## z_mu[4]    -0.92
## z_mu[5]     1.96
## sigma_a    4.74
## mu_a      91.45
## bL        1.53
## cL       -1.51
## mu_c      15.06
## z_sigma[1] -0.11
## z_sigma[2] 0.2
## z_sigma[3] 0.01
## z_sigma[4] -0.05
## z_sigma[5] -0.03
## sigma_c    0.44
## nobs       150
```

Table S7: Testing if non-linear relationships between latitude and the location summary statistics influence parameter estimates.

4. Literature cited

Ling SD, Johnson CR, Ridgway K, Hobday AJ, Haddon M (2009) Climate-driven range extension of a sea urchin: inferring future trends by analysis of recent population dynamics. Glob Change Biol 15:719-731

McElreath R (2020) rethinking: Statistical Rethinking book package. 2.01.
